# Optimizing insecticide deployment strategies to delay quantitative resistance in mosquito populations

**DOI:** 10.1007/s00285-026-02343-z

**Published:** 2026-02-23

**Authors:** Sylvère Kezeta-Bondja, Charles S. Wondji, Ramsès Djidjou-Demasse

**Affiliations:** 1 Centre for Research in Infectious Diseases, Yaoundé, Yaoundé, Cameroon; 2 Department of Mathematics, Faculty of Sciences, University of Yaounde I, Yaoundé, Yaoundé, Cameroon; 3 Department of Vector Biology, Liverpool School of Tropical Medicine, Liverpool, UK; 4 MIVEGEC, Univ. Montpellier, CNRS, IRD, Montpellier, France; 5 École Polytechnique de Thiès, Thiès, Sénégal France

**Keywords:** Nonlinear dynamical systems, Structured mathematical model, Quantitative insecticide resistance, Optimal control strategies, Adaptive insecticide management, 37N25, 35BXX, 92D15, 92D45

## Abstract

The large-scale use of insecticides remains a cornerstone of malaria vector control, but its long-term effectiveness is undermined by the evolution of quantitative insecticide resistance (qIR) in mosquito populations. We develop and analyze a mathematical model to identify optimal deployment strategies for two insecticides that differ only in their relative efficacy against target mosquito populations. Resistance is represented as a continuous phenotypic trait influencing mosquito fecundity and mortality, and the model accounts for successive deployment periods. Our results show that when mutational variance is high, the optimal strategy is to deploy the most effective insecticide at full coverage, regardless of its relative efficacy or pre-deployment exposure history. By contrast, when mutational variance is low, optimal deployment requires a transient reduction in coverage during early periods, with a threshold effect driven by both relative efficacy and initial exposure rates. Crucially, we find that, under the hypothesis that the first insecticide is ineffective against mosquitoes, simultaneous use of both insecticides is rarely optimal. Instead, sequential deployment–using one insecticide until resistance reaches a critical threshold, followed by optimal use of the second–delays resistance evolution and improves long-term control. These findings provide a theoretical foundation for adaptive qIR management strategies aimed at prolonging the effectiveness of insecticides in malaria vector control.

**Supplementary Information:**

The online version contains supplementary material available at 10.1007/s00285-026-02343-z.

## Introduction

Vector-borne diseases (VBDs) remain a major global health threat, causing over 700,000 deaths annually and affecting nearly 80% of the world’s population (World Health Organization 2017). Insecticide-based vector control remains one of the cornerstones of prevention, as emphasized in the Global Vector Control Response 2017–2030 (World Health Organization 2017). Among VBDs, malaria is particularly burdensome, especially in sub-Saharan Africa, with an estimated 263 million cases and 597,000 deaths in 2023 (WHO 2024). Control efforts have relied heavily on pyrethroid-based tools, notably insecticide-treated nets (ITNs) and indoor residual spraying (IRS), which accounted for roughly 78% of the malaria decline between 2000 and 2015 (Bhatt et al. 2015).

However, sustained pyrethroid use has driven widespread insecticide resistance (IR) in mosquito populations (Alout et al. 2017; World Health Organization 2018; Hemingway et al. 2016), undermining the effectiveness of current tools and compromising progress toward malaria elimination (World Health Organization 2012; Ranson and Lissenden 2016; Churcher et al. 2016; Killeen and Ranson 2018; Strode et al. 2014). Unlike agriculture, public health vector control has access to only a limited arsenal of insecticides (Ranson and Lissenden 2016), highlighting the urgent need for insecticide resistance management (IRM) strategies that can prolong insecticide efficacy (World Health Organization 2012). Overall, two main IRM approaches can be considered: rotation, in which insecticides with different modes of action are alternated over time, and mixture, in which different insecticides are applied simultaneously in the same location and at the same time.

The rapid rise of the IR has prompted numerous theoretical studies examining its evolutionary dynamics and the performance of IRM strategies. Many of these models, grounded in population genetics, assume a monogenic basis for IR, i.e., mutations in one or two genes confer resistance (Birget and Koella 2015; Levick et al. 2017; Gabrielkuniyoshi and Piodossantos 2017; Sudo et al. 2018; South and Hastings 2018; Mohammed-Awel and Gumel 2019, 2023). While such models have yielded important insights, they often overlook the transient evolutionary processes that precede fixation. Moreover, growing empirical evidence suggests that IR is frequently polygenic, with multiple genes contributing additively (Tatchou-Nebangwa et al. 2024; Weedall et al. 2020; Opondo et al. 2025). In this setting, IR can be viewed as a quantitative trait, represented by a continuous phenotypic value $x \in \Omega$, that influences mosquitoes life-history traits such as egg-laying (or fecundity) and mortality (Kezeta-Bondja et al. 2025).

Several studies compare the effectiveness of various IRM strategies, eg., (South and Hastings 2018; Helps et al. 2020; Madgwick and Kanitz 2022, 2024; Hobbs et al. 2023; Hobbs and Hastings 2025b, a). Collectively, these studies treated IR as a qualitative trait representing varying levels of reduced susceptibility and explore the evolutionary and operational dimensions of IRM, and provide complementary insights into how resistance genetics, spatial heterogeneity, and insecticide properties influence strategy effectiveness. In South and Hastings (2018); Helps et al. (2020), the authors account for selection pressure, genetic mixing during reproduction, and mutation, using these factors to compare the long-term performance of IRM. In Madgwick and Kanitz (2022, 2024), previous work is extended by incorporating spatial heterogeneity and mosquito dispersal between treated and untreated patches, with simulations assessing how different spatial deployment patterns affect both vector suppression and the spread of IR. Hobbs et al. (2023) developed a hybrid discrete–continuous model with seasonal forcing to investigate how environmental variability modifies IRM outcomes. Finally, (Hobbs and Hastings 2025a, b) incorporated stochasticity and more realistic genetic architectures (including partial dominance and non-additive effects) into continuous-trait models, highlighting the role of evolutionary uncertainty and trait variance in determining insecticide durability.

In line with the quantitative nature of the IR, here, we consider a scenario in which an initial insecticide ($\textrm{I}_{1}$) has lost effectiveness due to resistance, prompting the introduction of a second insecticide ($\textrm{I}_{2}$) with a specified relative efficacy compared to $\textrm{I}_{1}$. We develop an age-structured integro-differential model to describe the transient evolution of resistance in both exposed and unexposed adult mosquitoes. Three IRM strategies are evaluated for their ability to suppress the adult mosquito population: (i) simple rotation (denoted as Rot), in which insecticides $\textrm{I}_{1}$ and $\textrm{I}_{2}$ are alternated in successive update periods; (ii) simple mosaic (denoted as Mos), in which each cycle’s insecticide landscape is evenly divided, with 50% $\textrm{I}_{1}$ and 50% $\textrm{I}_{2}$; and (iii) optimal mosaic (denoted as Mos*), in which the proportions of $\textrm{I}_{1}$ and $\textrm{I}_{2}$ are optimized to minimize the mosquito population size over the deployment period. Building on earlier work on the quantitative nature of IR (Kezeta-Bondja et al. 2025), our study incorporates a continuous resistance trait within a structured modelling framework, thereby offering a novel tool for assessing both epidemiological and evolutionary outcomes.

The remainder of the paper is organized as follows. Section 2 describes the model formulation. Section 3 presents preliminary analytical results, including the existence of a unique maximal bounded semiflow and the conditions for steady states in the baseline model. Section 4 details parameterization and quantitative variables. Section 5 reports simulation outcomes, sensitivity analyses, and the influence of key parameters. Finally, Section 6 discusses the implications of our findings.

## The model description

The model captures the dynamics of a mosquito population under insecticide pressure. This population is captured by two developmental stages, with *E* representing the number of eggs and *A* the number of adult mosquitoes. Two types of insecticides, referred to as $\textrm{I}_{1}$ and $\textrm{I}_{2}$, are taken into account. At any given time *t*, the mosquito population is partitioned into three distinct groups: those unexposed to insecticides (represented by $E_0(t,x)$ for eggs and $A_0(t,a,x)$ for adults), those exposed to insecticide $\textrm{I}_{1}$ (denoted $E_1(t,x)$ and $A_1(t,a,x)$), and those exposed to insecticide $\textrm{I}_{2}$ (denoted $E_2(t,x)$ and $A_2(t,a,x)$). Here, $a\in \mathbb {R}_+$ indicates the age of adult mosquitoes, while $x\in \Omega \subset \mathbb {R}$ corresponds to their resistance level. A comprehensive list of the model’s variables and parameters is provided in Table 1.

**Table 1:** Model notations, state variables and parameters

**Model notations**		
Notation	Biological meaning (Unit)	
*t*	Chronological time	
*a*	Age of adult mosquito	
*x*	Level of mosquito insecticide resistance	
$\overline{T}$	Number of years of insecticide $\textrm{I}_{2}$ deployment	

**State variables**		
Variable	Description	
$E_{j}(t,x)$	Number of eggs laid by AFM in insecticide state *j* at time *t* and resistance level *x*	
$A_{j}(t,a,x)$	Number of adult mosquitoes in insecticide state *j*, aged *a* at time *t* with resistance level *x*	

**Fixed parameters**		
Notation	Biological meaning (Unit)	Value [Ref]
$\gamma _{j}(x)$	Proportion of eggs laid by AFM in insecticide state *j* that hatch (percentage/day)	0.75 [Assumed]
$\tau (x)$	Proportion of hatched eggs laid by AFM that reach adulthood (percentage/day)	0.67 Bayoh and Lindsay (2003)
$\mu _{j}(x)$	Natural death rate of eggs laid by AFM in insecticide state *j* (percentage/day)	0.2 [Assumed]
*A*	Adult mosquitoes age limit (in days)	30 Carnevale and Robert (2009)
$a_L$	Average age at which AFMs start laying eggs (in days)	3 Carnevale and Robert (2009)
$a_{LS}$	Average lifespan of Adult mosquitoes (in days)	21 Carnevale and Robert (2009)
$r_m$	Maximum number of eggs laid by the reference sensitive mosquito	200 CDC (2024)
$r_0$	Average number of eggs laid by the reference sensitive mosquito	$0.875 \times r_m$ [Assumed]
$r_1$	Average number of eggs laid by the reference resistant mosquito	$0.750 \times r_m$ [Assumed]

**Variable parameters**		
Notation	Biological meaning (Unit)	Range
$p_{\ell }$	Exposure rate to insecticide $\textrm{I}_{\ell }$ (dimensionless)	(0,1)
$\mathrm{r_{eff}}$	The relative effectiveness of insecticide $\textrm{I}_{2}$ compared to $\textrm{I}_{1}$ (dimensionless)	(0,1)
$m_{j}(x,y)$	Probability of mutation from IR level *y* to *x* for AFM in insecticide state *j* (dimensionless)	defined by (4.1)
*r*(*a*, *x*)	Egg-laying rate for an AFM (number/day)	defined by (4.2)
$d_{j}(a,x)$	Natural death rate of adult mosquitoes in insecticide state *j* (percentage/day)	defined by (4.3)-(4.4)
$p_{1}^{S}$	Probability of surviving $\textrm{I}_{1}$-insecticide exposure during one day	(0,1)
$p_{2}^{S}$	Probability of surviving $\textrm{I}_{2}$-insecticide exposure during one day	defined by (4.6)
$\textrm{var}_0$	Mutational variance within unexposed AFM population	$\{10^{-2} ; 2\times 10^{-3} ; 10^{-3} \}$
$\textrm{var}$	Mutational variance ratio	$\{2 ; 3 \}$
$\textrm{var}_\ell$	Mutational variance of $\textrm{I}_{\ell }$ exposed AFM population	$\textrm{var}_\ell =\textrm{var}\times \textrm{var}_0$
$\bar{d}_{0,\ell }$	Death rate of reference sensitive mosquito due to $\textrm{I}_{\ell }$ exposure	$- \log (p_{\ell }^{S})$
$\bar{d}_{\ell }$	Death rate of reference $\textrm{I}_{\ell }$ resistant mosquito due to $\textrm{I}_{\ell }$ exposure	$- \log (1-p_{\ell }^{S})$
$\kappa$	Scaling positive constant	(0,1)

AFM=Adult Female Mosquito , $j\in \{0,1,2\}$ , $\ell \in \{1,2\}$

### Baseline model with a spatially uniform insecticide landscape

We define the following aggregate quantity:

$$\begin{aligned} E(t)= \sum _{j=0}^{2} \int _\Omega E_{j}(t,x) \textrm{d}x , \end{aligned}$$

representing the total eggs population at time *t*. The reproductive capacity of the population is assumed to be regulated by a density-dependent function *H*(*E*(*t*)), given by:

2.1 $$\begin{aligned} H(E(t))= \left( 1 + E(t) \right) ^{-\kappa }, \end{aligned}$$

where the constant $\kappa > 0$ modulates the strength of density dependence.

Eggs laid by adult female mosquitoes (AFMs) with resistance level *x* experience mortality at rate $\mu _j(x)$ and hatch at rate $\gamma _j(x)$, where $j \in \{0,1,2\}$ denotes unexposed mosquitoes ($j=0$), mosquitoes exposed to $\textrm{I}_{1}$ ($j=1$), and mosquitoes exposed to $\textrm{I}_{2}$ ($j=2$). Of the hatched eggs, only a fraction $\tau (x)$ emerges as adult mosquitoes.

We assume that the insecticide present in the environment, although affecting the life-history traits of the different developmental stages of the mosquito, does not induce mortality in eggs or larvae. Instead, it directly increases the mortality of adult mosquitoes–i.e., vectors that have emerged from the aquatic developmental stage. Consequently, insecticides $$\textrm{I}_{1}$$ and $$\textrm{I}_{2}$$ are present in the environment such that newly emerged adult vectors may be exposed to $$\textrm{I}_{1}$$ with probability $$p_1$$ or to $$\textrm{I}_{2}$$ with probability $$p_2$$. Under the assumption that an adult mosquito cannot be simultaneously exposed to both insecticides, ($$ p_1 + p_2,$$) represents the probability that an emerging vector is exposed to either $$\textrm{I}_{1}$$ or $$\textrm{I}_{2}$$, and thus ($$1-p_1-p_2$$) represents the probability that an emerging vector escapes exposure to both $$\textrm{I}_{1}$$ and $$\textrm{I}_{2}$$.

The boundary conditions for the adult compartments at age zero are thus:

2.2 $$\begin{aligned} {\left\{ \begin{array}{ll} A_{0}(t,a=0,x)= \left( 1 - p_{1} - p_{2}\right) \tau (x) \left[ \gamma _{0}(x)E_{0}(t,x) + \gamma _{1}(x)E_{1}(t,x) + \gamma _{2}(x)E_{2}(t,x) \right] , \\ A_{1}(t,a=0,x)= p_{1} \tau (x) \left[ \gamma _{0}(x)E_{0}(t,x) + \gamma _{1}(x)E_{1}(t,x) + \gamma _{2}(x)E_{2}(t,x) \right] , \\ A_{2}(t,a=0,x)= p_{2} \tau (x) \left[ \gamma _{0}(x)E_{0}(t,x) + \gamma _{1}(x)E_{1}(t,x) + \gamma _{2}(x)E_{2}(t,x) \right] . \end{array}\right. } \end{aligned}$$

With these definitions, the model dynamics with a spatially uniform insecticide landscape is governed by the following age-structured integro-differential equations, for $j\in \{0,1,2\}$:

2.3 $$\begin{aligned} {\left\{ \begin{array}{ll} \partial _t E_{j}(t,x)= H(E(t)) \int _{\Omega } \int _0^\infty m_{j}(x,y)r(a,y)A_{j}(t,a,y)\textrm{d}a\textrm{d}y - (\mu _{j}(x) + \gamma _{j}(x)) E_{j}(t,x), \\ \left( \partial _t + \partial _a \right) A_{j}(t,a,x)= - d_{j}(a,x) A_{j}(t,a,x), \end{array}\right. } \end{aligned}$$

where the death rates of adult mosquitoes are denoted by $d_j(a,x)$, for $j \in \{0,1,2\}$. The term

$$ H(E(t))\int _{\Omega } \int _0^\infty m_j(x,y) r(a,y) A_j(t,a,y) \textrm{d}a \textrm{d}y $$

describes the rate at which eggs with resistance level *x* are produced by AFMs of resistance level *y* and age *a* in insecticide state *j*. The function $m_j(x,y)$ encodes the probability of mutation from resistance level (or phenotype) $y$ to resistance level (or phenotype) $x$. Thus, mutations randomly displace strains in the phenotype space at each eggs-laying generation according to the mutation kernel $m_j$. The function $r(a,y)$ denotes the age-specific oviposition rate.

The system is complemented by the following initial conditions:

$$ E_{j}(0,x)= E_{j,0}(x), \quad A_{j}(0,a,x)= A_{j,0}(a,x). $$

### The model with a variable insecticide landscape

Model (2.2)–(2.3) is based on the assumption of a spatially and temporally uniform insecticide landscape. However, to better capture field conditions, we now consider a more general framework allowing for landscape variability. We assume that during the $\overline{T}$ years of insecticide deployment period, the composition of the insecticide landscape (comprising $\textrm{I}_{1}$ and $\textrm{I}_{2}$) is revised *N* times at regular intervals of length $\mathrm{D_{ur}}$, at times $T_0=0$, $T_1$, $\cdots$, $T_{N-1}$, such that $T_n= n \times \mathrm D_{ur}$, for $n=0, \cdots , (N-1)$, with $N= \overline{T}/ \mathrm D_{ur}$. Therefore, during each period $[T_{n-1}, T_n]$, with $n=1, \cdots , N$, the insecticide landscape composition is defined by the proportions $p^n_1$ and $p^n_2$, representing the proportion of insecticides $\textrm{I}_{1}$ and $\textrm{I}_{2}$, respectively, with $p^n_1+p^n_2\le 1$. Consistent with previous notations, for $n \in \{ 1, \cdots , N\}$, $E_{j}^{n}(t,\cdot )=A_j(t,\cdot )$ and $A_{j}^{n}(t,\cdot ,\cdot )=A_j(t,\cdot ,\cdot )$, for $t\in [T_{n-1},T_{n})$. We then have, for $n \in \{ 1, \cdots , N\}$ and $t\in [T_{n-1},T_{n})$:

2.4 $$\begin{aligned} {\left\{ \begin{array}{ll} A_{0}^{n}(t,a=0,x)= \left( 1 - p_{1}^{n} - p_{2}^{n}\right) \tau (x) \left[ \gamma _{0}(x)E_{0}^{n}(t,x) + \gamma _{1}(x)E_{1}^{n}(t,x) + \gamma _{2}(x)E_{2}^{n}(t,x) \right] , \\ A_{1}^{n}(t,a=0,x)= p_{1}^{n} \tau (x) \left[ \gamma _{0}(x)E_{0}^{n}(t,x) + \gamma _{1}(x)E_{1}^{n}(t,x) + \gamma _{2}(x)E_{2}^{n}(t,x) \right] , \\ A_{2}^{n}(t,a=0,x)= p_{2}^{n} \tau (x) \left[ \gamma _{0}(x)E_{0}^{n}(t,x) + \gamma _{1}(x)E_{1}^{n}(t,x) + \gamma _{2}(x)E_{2}^{n}(t,x) \right] , \end{array}\right. } \end{aligned}$$

and for $$j\in \{0,1,2\}$$:

2.5 $$\begin{aligned} {\left\{ \begin{array}{ll} \partial _t E_{j}^{n}(t,x)= H(E^{n}(t)) \int _{\Omega } \int _0^\infty m_{j}(x,y)r(a,y)A_{j}^{n}(t,a,y)\textrm{d}a\textrm{d}y - (\mu _{j}(x) + \gamma _{j}(x)) E_{j}^{n}(t,x), \\ \left( \partial _t + \partial _a \right) A_{j}^{n}(t,a,x)= - d_{j}(a,x) A_{j}^{n}(t,a,x), \end{array}\right. } \end{aligned}$$

with the initial conditions $E_{j}^1(0,x) = E_{j,0}(x),$ $A_{j}^1(0,a,x) = A_{j,0}(a,x)$ and $E^n_j(T_{n-1},x) = E^{n-1}_j(T_{n-1},x),$ $A^n_j(T_{n-1},a,x) = A^{n-1}_j(T_{n-1},a,x)$ when $n \in \{ 2, 3, \ldots , N\}$.

Systems (2.2)–(2.3) and (2.4)-(2.5) will be considered under the following assumptions:

#### Assumption 2.1

For $$j \in \{0, 1, 2\}$$,

1. $\tau$, $\mu _{j}$ and $\gamma _{j}$ are positive, continuous and bounded functions on $\Omega$.
2. The functions *r* and $d_{j}$ are positive, continuous and bounded on $(0,\infty ) \times \Omega$.
3. The mutation kernel $m_{j}$, is strictly positive almost everywhere, Lipschitz continuous, integrable, bounded on $\Omega \times \Omega$, and has a unit mass, ie., $\int _{\Omega } m_{j}(x,y)\textrm{d}x = 1$ for $y\in \Omega$.

#### Assumption 2.2

The mutation kernel $m_{j}, j \in \{0, 1, 2\}$,

1. is symmetric on $\Omega \times \Omega$, *ie.*, $m_{j}(x,y) = m_{j}(y,x)$.
2. decays rather rapidly towards infinity in the sense that $\displaystyle \lim _{|x| \rightarrow \infty } |x|^\ell m_j(x,\cdot ) = 0$, for all $\ell \in \mathbb {N}$.

## Preliminary results under constant insecticide exposure rate

This section is devoted to the main results of Model (2.2)–(2.3). Our analysis reveals the existence of a unique maximal bounded semiflow, a significant result that underscores the underlying dynamics of System (2.2)–(2.3) under constant exposure. Furthermore, we provide the necessary and sufficient conditions for the existence of equilibria for System (2.2)–(2.3) under constant exposure. The autonomous nature of System (2.2)–(2.3) facilitates the derivation of these analytical results just as rewriting System (2.2)–(2.3) into a compact form.

By setting, $u(t,x)= \left( E_{0}(t,x), E_{1}(t,x), E_{2}(t,x)\right)$ and $v(t,a,x)= (A_{0}(t,a,x), A_{1}(t,a,x), A_{2}(t,a,x))$, Model (2.2)–(2.3) rewrites as:

3.1 $$\begin{aligned} {\left\{ \begin{array}{ll} \displaystyle \partial _t u(t,x) = h(u)(t) \int _{\Omega } \int _0^{\infty } m(x,y) r(a,y) v(t,a,y) \textrm{d}a \textrm{d}y - \mathcal {N}(x) u(t,x),\\ \displaystyle v(t,a=0,x) = \mathcal {P} \mathcal {C}(x) u(t,x),\\ \displaystyle (\partial _t+\partial _a) v(t,a,x) = - \mathcal {D}(a,x)v(t,a,x). \end{array}\right. } \end{aligned}$$

with,

$$ \begin{aligned}&m(x,y)=\textrm{diag}(m_{j}(x,y))_j, \quad \mathcal {N}(x)=\textrm{diag}(\mu _{j}(x)+\gamma _{j}(x))_j, \\&\mathcal {D}(a,x)=\textrm{diag}(d_{j}(a,x))_j,\\&\mathcal {P}= \textrm{diag} \left( 1 -p_{1} -p_{2}, p_1, p_2 \right) ,\quad \mathcal {C}(x) = \tau (x) \begin{pmatrix} \gamma _{0}(x) & \quad \gamma _{1}(x) & \quad \gamma _{2}(x)\\ \gamma _{0}(x) & \quad \gamma _{1}(x) & \quad \gamma _{2}(x)\\ \gamma _{0}(x) & \quad \gamma _{1}(x) & \quad \gamma _{2}(x)\\ \end{pmatrix}, \end{aligned} $$

and, where

3.2 $$\begin{aligned} h(u)(t)= H(E(t)) = \left( 1+ \bar{h}(u)(t) \right) ^{-\kappa }, \quad \text {with} \quad \bar{h}(u)(t)= E(t). \end{aligned}$$

### Semiflow generated by System (2.2)–(2.3)

To establish the global well-posedness, positivity, and dissipativity of the solutions of System (2.2)–(2.3), or equivalently of (3.1), we formulate it as an abstract Cauchy problem. Let us introduce the Banach space $X = L^{1}(\Omega ,\mathbb {R}^3) \times L^{1}(\Omega ,\mathbb {R}^3) \times L^{1}((0,\infty ) \times \Omega ,\mathbb {R}^3)$, endowed with the usual product norm $\Vert \cdot \Vert _X$, as well as its positive cone $X_+ = L_{+}^{1}(\Omega ,\mathbb {R}^3) \times L_{+}^{1}(\Omega ,\mathbb {R}^3) \times L_{+}^{1}((0,\infty ) \times \Omega ,\mathbb {R}^3)$. Consider the linear operator $\mathcal {A}: D(\mathcal {A}) \subset X \rightarrow X$ defined by $D(\mathcal {A}) = W^{1,1}(\Omega ,\mathbb {R}^3) \times \{0_{L^{1}(\Omega ,\mathbb {R}^3)}\} \times W^{1,1}((0,\infty ) \times \Omega ,\mathbb {R}^3)$ and

3.3 $$\begin{aligned} \mathcal {A} \begin{pmatrix} u \\ {0}_{L^{1}} \\ v \end{pmatrix} = \begin{pmatrix} - \mathcal {N} u \\ - v(0,\cdot ) \\ - \partial _a v - \mathcal {D}v \end{pmatrix}. \end{aligned}$$

Note that $\mathcal {A}$ is not densely defined in *X* as $\overline{D(\mathcal {A})} = X_{0} \subset X$. We note $X_{0+} = X_{0} \cap X_{+}$ the positive cone of $X_{0}$. Let $F: X_0 \rightarrow X$ be the non-linear map defined by:

3.4 $$\begin{aligned} F \begin{pmatrix} u \\ 0_{L^{1}} \\ v \end{pmatrix} = \begin{pmatrix} h(u) \int _{\Omega } \int _0^{\infty } m(\cdot ,y) r(a,y) v(\cdot ,a,y) \textrm{d}a \textrm{d}y \\ \mathcal {P}\mathcal {C} u \\ 0_{L^{1}((0,\infty ) \times \Omega ,\mathbb {R}^3)} \end{pmatrix}. \end{aligned}$$

By setting $z(t) = (u(t,\cdot ), 0_{L^{1}}, v(t,\cdot ,\cdot ))^T$ and $z_0 = (u_0, 0_{L^{1}}, v_0)^T$ the associated initial condition, System (2.2)–(2.3) rewrites as the following abstract Cauchy problem:

3.5 $$\begin{aligned} {\left\{ \begin{array}{ll} \displaystyle \frac{\textrm{d}z(t)}{\textrm{d}t} = \mathcal {A}z(t) + F(z(t)), \quad t > 0 \\ z(0) = z_{0}. \end{array}\right. } \end{aligned}$$

We have the following result.

#### Theorem 3.1

Let Assumption 2.1 be satisfied. There exists a unique strongly continuous semiflow $\{T(t,\cdot ): X_{0} \rightarrow X_{0}\}_{t \ge 0}$ such that, for each $z_{0} \in X_{0+}$, the map $z \in \mathcal {C}\left( (0, \infty ), X_{0+} \right)$ defined by $z = T(\cdot , z_{0})$ is a mild solution of (3.5), *ie.*, $\displaystyle \int _0^t z(s)\textrm{d}s \in X_{0}$ and $\displaystyle z(t) = z_{0} + \mathcal {A}\int _0^t z(\sigma )\textrm{d}\sigma + \int _0^t F(z(\sigma ))\textrm{d}\sigma$ for all $t \ge 0$. Moreover the semiflow $\{ T(t, \cdot )\}_{t \ge 0}$ satisfies the following properties:

1. Let $T(t,z_{0}) = (u(t,\cdot ), 0_{L^1}, v(t,\cdot ,\cdot ))^T$, then the following Volterra formulation holds true
  $$ \boldsymbol{v}(t,a,x) = {\left\{ \begin{array}{ll} \boldsymbol{\Pi }(a-t,a,x)\,v_0(a-t,x) \quad & \text {if}\ \ t\le a,\\ \boldsymbol{\Pi }(0,a,x)\,\mathcal {P}\mathcal {C}(x)\,u(t-a,x)& \text {if}\ \ t> a, \end{array}\right. } $$
  coupled with the *u*-equation of (3.1), and where, for $0\le \alpha \le \beta$,
  3.6 $$\begin{aligned} \boldsymbol{\Pi }(\alpha ,\beta ,x)=\textrm{diag}\left( \pi _{j}(\alpha ,\beta ,x) \right) _j, \quad \text {with} \quad \pi _{j}(\alpha ,\beta ,x):= e^{-\int _{\alpha }^{\beta }d_{j}(\sigma ,x)\textrm{d}\sigma }. \end{aligned}$$
2. For all $z_{0}\in X_0$, we have
  $$ E(t) \le H^{-1} \left( \frac{\bar{c}\beta }{\nu } \right) ,\quad \forall t>0 $$
  and
  $$ \sum _{j=0}^{2} \int _\Omega \frac{1}{\tau (x)} \int _0^\infty A_j(t,a,x) \textrm{d}a \textrm{d}x \le w_0 e^{-t\beta }+ \frac{\alpha }{\beta } H^{-1} \left( \frac{\bar{c}\beta }{\nu } \right) \left( 1- e^{-t\beta }\right) , \quad \forall t>0 $$
  where $w_0= \sum _{j=0}^{2} \int _\Omega \frac{1}{\tau (x)} \int _0^\infty A_{j,0}(a,x) \textrm{d}a \textrm{d}x$. The constants $\nu$, $\alpha$, and $\beta$ are defined by: $\nu = \Vert r\Vert _{\infty } \max _{j\in \{0,1,2\}} \Big \{ \int _{\Omega } \sup _y m_{j}(x,y) \textrm{d}x \Big \}$, $\alpha =\max _{ j\in \{0,1,2\}} \left\{ \Vert \gamma _j\Vert _{\infty } \right\}$, $\beta = \frac{\min _{j \in \mathcal {J}} \left\{ \inf _{(a,x) \in \mathbb {R}_+\times \Omega } d_j \right\} }{\max _{j \in \mathcal {J}} \left\{ \Vert \tau \Vert _{\infty } \right\} }$. The constant $\bar{c}$ satisfies: $0< \bar{c} < \min \left( \frac{\min _{j \in \{0,1,2\}}\left( \underline{\mu }_j+ \underline{\gamma }_j \right) }{ \alpha }, \frac{\nu }{\beta } \right)$. Here, $\underline{f}:= \inf \limits _{x \in \Omega }f$.
3. The semiflow $\{T(t,\cdot ): X_{0} \rightarrow X_{0}\}_{t \ge 0}$ is bounded dissipative, *ie.*, there exists a bounded set $B\subset X_0$ that attracts any bounded set $\mathcal {R} \subset X_0$, basically there exists $\zeta =\zeta (\mathcal {R},B)\ge 0$ such that $T(t,\mathcal {R})\subset B$ for $t\ge \zeta$.
4. The semiflow $\{T(t,\cdot )\}_t$ is asymptotically smooth in $X_+$, *ie.*, for any nonempty, closed, bounded and positively invariant set $B\subset X_+$, there exists a compact set $\mathcal {R}\subset X_+$ such that $\lim _{t\rightarrow \infty }d_h(T(t,B), \mathcal {R})=0$, where $d_h$ is the Hausdorff semi-distance defined as in Hale (2014).

#### Proof of Theorem 3.1

It is straightforward to verify that the operator $\mathcal {A}$ defined in (3.3) is a Hille–Yosida operator, and that the nonlinear map *F* defined in (3.4) is positive, continuous, and locally Lipschitz on $X_0$. Standard semigroup theory then ensures the existence and uniqueness of a mild solution to (3.5) (see (Magal and Ruan 2018; Thieme 2011, 1990)). The equivalent Volterra formulation is also well-established; see, e.g., (Iannelli 1995; Webb 1985). The proof of Theorem 3.1 closely follows that of Theorem 3.1 in Kezeta-Bondja et al. (2025).

For the boundedness, let us set

$$\begin{aligned} E(t)= \sum _{j=0}^{2} \int _\Omega E_{j}(t,x) \textrm{d}x, \quad A(t)= \sum _{j=0}^{2} \int _\Omega \frac{1}{\tau (x)} \int _{\mathbb {R}_+} A_j(t,a,x) \textrm{d}a \textrm{d}x. \end{aligned}$$

Then, by (2.2)-(2.3), it comes

$$\begin{aligned} \dot{E}(t)=&H(E(t)) \sum _{j=0}^{2} \int _{\Omega } \int _{\Omega }\int _{\mathbb {R}^+}m_j(x,y) r(a,y) A_j(t,a,y) \textrm{d}a\textrm{d}y \textrm{d}x \\&- \sum _{j=0}^{2} \int _{\Omega } (\mu _j(x)+ \gamma _j(x)) E_{j}(t,x) \textrm{d}x , \\ \dot{A}(t)=&\sum _{j=0}^{2} \int _{\Omega } \gamma _j(x) E_j(t,x) \textrm{d}x - \int _{\Omega } \int _{\mathbb {R}_+} \frac{d_j(a,x)}{\tau (x)} A_j(t,a,x) \textrm{d}a \textrm{d}x . \end{aligned}$$

By Assumption 2.1, we find that

3.7 $$\begin{aligned} \dot{E}(t) \le&\nu H\left( E(t)\right) A(t) - \zeta E (t) , \nonumber \\ \dot{A}(t) \le&\alpha E(t) - \beta A(t), \end{aligned}$$

where $\zeta = \min _{j \in \{0,1,2\}}\left( \underline{\mu }_j+ \underline{\gamma }_j \right)$, $\nu = \Vert r\Vert _{\infty } \max _{j\in \{0,1,2\}} \Big \{ \int _{\Omega } \sup _y m_{j}(x,y) \textrm{d}x \Big \}$, $\alpha =\max _{j \in j\in \{0,1,2\}} \left\{ \Vert \gamma _j\Vert _{\infty } \right\}$, and $\beta = \frac{\min _{j \in \mathcal {J}} \left\{ \inf _{(a,x) \in \mathbb {R}_+\times \Omega } d_j \right\} }{\max _{j \in \mathcal {J}} \left\{ \Vert \tau \Vert _{\infty } \right\} }$. Here, $\underline{f}:= \inf \limits _{x \in \Omega }f$.

Let $\bar{c} >0$, and set $W= E+ \bar{c} A$. Estimates (3.7) give

3.8 $$\begin{aligned} \dot{W}(t) \le \left( \bar{c} \alpha - \zeta \right) E(t)+ \left( \nu H\left( E(t)\right) -\bar{c}\beta \right) A(t). \end{aligned}$$

Since the function *H*, introduced in (2.1), is decreasing and takes values in (0, 1), we have

$$ \nu H\left( E(t)\right) - \bar{c} \beta <0 \quad \text { iff } \quad E(t) > H^{-1} \left( \frac{\bar{c}\beta }{\nu } \right) , $$

where, of course, the above estimate holds on the necessary condition $\frac{\bar{c} \beta }{\nu } \in (0,1)$. Thus, by choosing $\bar{c}$, such that

3.9 $$\begin{aligned} 0< \bar{c} < \min \left( \frac{\zeta }{ \alpha }, \frac{\nu }{\beta } \right) , \end{aligned}$$

estimate (3.8) leads to $\dot{W}(t)<0$ as soon as $E(t) > H^{-1} \left( \frac{\bar{c}\beta }{\nu } \right)$. From where we find that *E* is ultimately bounded and, for all *t*,

$$ E(t) \le H^{-1} \left( \frac{\bar{c}\beta }{\nu } \right) , $$

with $$\bar{c}$$ satisfying (3.9).

Finally, by (3.7), we find for all $t\ge 0$:

$$ A(t) \le A(0) e^{-t\beta }+ \frac{\alpha }{\beta } H^{-1} \left( \frac{\bar{c}\beta }{\nu } \right) \left( 1- e^{-t\beta }\right) . $$

$$\square $$

Still under a constant insecticide exposure rate in System (2.2)–(2.3), other technical results–such as the asymptotic behavior and uniform persistence–can also be investigated. These analyses, however, lie beyond the primary scope of this study. They can nevertheless be addressed by closely following the methodology presented in (Kezeta-Bondja et al. 2025, Section 4).

### Steady states of System (2.2)–(2.3)

We now turn to the existence of steady states for System (2.2)–(2.3), or equivalently, for the abstract formulation (3.5). The system always admits a mosquito-free steady state, denoted by $\mathcal {E}^0=0$. Additional non-trivial steady states may also exist under suitable conditions.

Let us set, for all $x\in \Omega$ and $i,j\in \{0,1,2\}$:

3.10 $$\begin{aligned} \Gamma _{i,j}(x)= \frac{\tau (x)\gamma _{j}(x)}{\mu _{j}(x) + \gamma _{j}(x)} \int _0^\infty r(a,x) \pi _{i}(0,a,x) \textrm{d}a. \end{aligned}$$

Note that $\Gamma _{i,j}(x)$– hereafter referred to as the *fitness function*–represents the reproductive number of AFMs with insecticide resistance level *x* in insecticide state *j*, given that they were previously in insecticide state *i*. In the above expression, the survival probability $\pi _{i}(0, a, x)$, introduced in (3.6), is the likelihood that an AFM individual with IR level *x* survives up to age *a*. When multiplied by *r*(*a*, *x*), it gives the individual’s contribution to egg production at age *a*. Integrating this product over all ages yields the total number of eggs produced during the individual’s lifetime. Note that, under Assumption 2.1, the functions $\Gamma _{i,j}: \Omega \mapsto \mathbb {R}_+$ are positive and continuous.

When $i=j$, we simplify the notation by writing $\Gamma _j= \Gamma _{j,j},$ which represents the fitness of an AFM individual within the *j*-insecticide state.

The existence of steady states of System (2.2)–(2.3) are closely linked to the spectral properties of linear operators $\mathcal {L}_j: L_{+}^{1}(\Omega , \mathbb {R}^2) \rightarrow L_{+}^{1}(\Omega , \mathbb {R}^2)$ and $\mathcal {L}:L_{+}^{1}(\Omega , \mathbb {R}^3) \rightarrow L_{+}^{1}(\Omega , \mathbb {R}^3)$ defined by :

$$\begin{aligned} \mathcal {L}_j[\varphi ](\cdot )= \int _{\Omega } \textrm{diag} \left( (1 -p_{j})m_0(\cdot ,y), p_j m_j(\cdot ,y) \right) \left( \Gamma _{i,\ell }(y) \right) _{i,\ell \in \{0,j\}} \varphi (y)\textrm{d}y, \quad j\in \{1,2\}, \end{aligned}$$

and

3.11 $$\begin{aligned} \mathcal {L}[\phi ](\cdot )= \mathcal {P} \int _{\Omega } {m}(\cdot , y) \left( \Gamma _{i,j}(y) \right) _{i,j\in \{0,1,2\}} \phi (y)\textrm{d}y. \end{aligned}$$

Under Assumptions 2.1 and 2.2, note that the linear operators $\mathcal {L}_j$, in addition to being positive, are also compact and irreducible (see (Djidjou-Demasse et al. 2017, Theorem 4.1)). Therefore, by the Krein–Rutman theorem, the spectral radius $\textrm{r}(\mathcal {L}_j)$ of $\mathcal {L}_j$ is positive, and the associated eigenfunction is strictly positive. The following result is then obtained.

#### Theorem 3.2

Let Assumptions 2.1 and 2.2 be satisfied. Then,

1. When $p_1 = 0$ and $\textrm{r}(\mathcal {L}_2)>1$, System (2.2)–(2.3) admits a steady state $\mathcal {E}_1= \left( E_j^*, A_j^*\right) _{j=0,1,2}$ that is free of mosquitoes exposed to insecticide $\textrm{I}_{1}$, such that: $E_1\equiv 0$, $A_1\equiv 0$, and
  $$ \begin{aligned}&E_j^*= \left( \left( \textrm{r}(\mathcal {L}_2)\right) ^{1/\kappa }-1\right) C_\textrm{cte2}^{-1} (\mu _j+\gamma _j)^{-1}\psi _2^j, \quad j=0,2, \\&A_0^*(a,x) = (1-p_2) \left( \left( \textrm{r}(\mathcal {L}_2)\right) ^{1/\kappa }-1\right) C_\textrm{cte2}^{-1} \tau (x) \pi _0(0,a,x) \sum _{j=0,2} \frac{\gamma _j}{\mu _j+\gamma _j}\psi _2^j (x),\\&A_2^*(a,x) = p_2 \left( \left( \textrm{r}(\mathcal {L}_2)\right) ^{1/\kappa }-1\right) C_\textrm{cte2}^{-1} \tau (x) \pi _2(0,a,x) \sum _{j=0,2} \frac{\gamma _j}{\mu _j+\gamma _j}\psi _2^j (x), \end{aligned} $$
  where $\psi _2=(\psi _2^j)_{j=0,2}>0$ is the eigen vector associated to $\textrm{r}(\mathcal {L}_2)>1$, and $C_\textrm{cte2}=C_\textrm{cte2}(\psi _2)$ is a positive constant define later by (3.20).
2. When $p_2 = 0$ and $\textrm{r}(\mathcal {L}_1)>1$, System (2.2)–(2.3) has a steady state $\mathcal {E}_2= \left( E_j^*, A_j^*\right) _{j=0,1,2}$ that is free of mosquitoes exposed to insecticide $\textrm{I}_{2}$, such that: $E_2\equiv 0$, $A_2\equiv 0$, and
  $$ \begin{aligned}&E_j^*= \left( \left( \textrm{r}(\mathcal {L}_1)\right) ^{1/\kappa }-1\right) C_\textrm{cte1}^{-1} (\mu _j+\gamma _j)^{-1}\psi _1^j, \quad j=0,1, \\&A^*_0(a,x) = (1-p_1) \left( \left( \textrm{r}(\mathcal {L}_1)\right) ^{1/\kappa }-1\right) C_\textrm{cte1}^{-1} \tau (x) \pi _0(0,a,x) \sum _{j=0}^1 \frac{\gamma _j}{\mu _j+\gamma _j}\psi _1^j(x),\\&A^*_1(a,x) = p_1 \left( \left( \textrm{r}(\mathcal {L}_1)\right) ^{1/\kappa }-1\right) C_\textrm{cte1}^{-1} \tau (x) \pi _1(0,a,x) \sum _{j=0}^1 \frac{\gamma _j}{\mu _j+\gamma _j}\psi _1^j(x), \end{aligned} $$
  with $\psi _1=(\psi _1^j)_{j=0,1}>0$ is the eigenfunction associated to $\textrm{r}(\mathcal {L}_1)>1$, and $C_\textrm{cte1}=C_\textrm{cte1}(\psi _1)$ is a positive constant define later by (3.21).
3. When $p_1,p_2 \in (0,1)$ and $\textrm{r}(\mathcal {L}) > 1$, System (2.2)–(2.3) possess a steady state $\mathcal {E}^{*}= (E_{j}^*,A_{j}^*)_{j=0,1,2}$, with mosquitoes exposed to both insecticides, such that
  $$ \begin{aligned}&E_j^*= \left( \left( \textrm{r}(\mathcal {L})\right) ^{1/\kappa }-1\right) C_\textrm{cte}^{-1} (\mu _j+\gamma _j)^{-1}\psi ^j, \quad j=0,1,2, \\&A^*_j (a,x)= \mathcal {P}_{j,j} \left( \left( \textrm{r}(\mathcal {L})\right) ^{1/\kappa }-1\right) C_\textrm{cte}^{-1}\tau (x) \pi _j(0,a,x) \sum _{j=0}^2\frac{\gamma _j}{\mu _j+\gamma _j}\psi ^j(x), \quad j=0,1,2, \end{aligned} $$
  where $\psi =(\psi ^j)_{j=0,1,2}>0$ is the eigenfunction associated to $\textrm{r}(\mathcal {L})>1$, and $C_\textrm{cte}=C_\textrm{cte}(\psi )$ is a positive constant define later by (3.22).

The next result proceed further towards a more explicit expression of the spectral radii $\textrm{r}(\mathcal {L}_j)$ and $\textrm{r}(\mathcal {L})$ of the operators $\mathcal {L}_j$ and $\mathcal {L}$.

#### Theorem 3.3

Let Assumptions 2.1 and 2.2 be satisfied. Additionally, assume that $\gamma _\ell = \gamma$ and $\mu _\ell = \mu$, for all $\ell \in \{0,1,2\}$. Then, the spectral radii $\textrm{r}(\mathcal {L}_j)$ and $\textrm{r}(\mathcal {L})$ of the operators $\mathcal {L}_j$, with $j=1,2$, and $\mathcal {L}$ can be more explicitly expressed as:

$$ \textrm{r}(\mathcal {L}_j)= \textrm{r}(\mathcal {M}_j), \text { for j=1,2,} \quad \text {and} \quad \textrm{r}(\mathcal {L})= \textrm{r}(\mathcal {M}), $$

with linear operators $\mathcal {M}_j:L^1(\Omega ,\mathbb {R})\rightarrow L^1(\Omega ,\mathbb {R})$ and $\mathcal {M}:L^1(\Omega ,\mathbb {R})\rightarrow L^1(\Omega ,\mathbb {R})$ given by

$$ \begin{aligned}&\mathcal {M}_j\varphi = \int _{\Omega } \left[ m_0(\cdot , y) (1-p_j) \Gamma _{0}(y) +m_j(\cdot , y) p_j \Gamma _{j}(y)\right] \varphi (y)\textrm{d}y, \\&\mathcal {M}\varphi = \int _{\Omega } \left[ m_0(\cdot , y) (1-p_1-p_2) \Gamma _{0}(y) +m_1(\cdot , y) p_1 \Gamma _{1}(y) +m_2(\cdot , y) p_2 \Gamma _{2}(y)\right] \varphi (y)\textrm{d}y. \end{aligned} $$

#### Remark 3.4

The existence of steady states of System (2.2)-(2.3) are linked to the spectral radii $\textrm{r}(\mathcal {L}_j)$ and $\textrm{r}(\mathcal {L})$ of the operators $\mathcal {L}_j$ and $\mathcal {L}$, for mutation kernels $m_j$’s satisfying Assumptions 2.1 and 2.2. In particular, when considering a family of mutation kernels $m_j^\zeta$ that depend on a small positive parameter $\zeta$ (with $\zeta \ll 1$), $\textrm{r}(\mathcal {L}_j)$’s and $\textrm{r}(\mathcal {L})$ can be directly approximated from the fitness functions $\Gamma _j$’s. Indeed, assume that

$$\begin{aligned} m_j^\zeta (x) = \zeta ^{-1} m_j\left( \zeta ^{-1} x \right) , \end{aligned}$$

where $\zeta \ll 1$ captures the variance of mutations across the phenotypic space.

Let us set

3.12 $$\begin{aligned} \begin{aligned}&\Theta _{[p_1,p_2]}= (1-p_1-p_2) \Gamma _{0} + p_1 \Gamma _{1} + p_2 \Gamma _{2}. \end{aligned} \end{aligned}$$

The function $\Theta _{[p_1,p_2]}$ denotes the fitness of a mosquito in a mixed landscape where insecticides $\textrm{I}_{1}$ and $\textrm{I}_{2}$ are present in proportions $p_1$ and $p_2$, respectively.

We then introduce the sets

3.13 $$\begin{aligned} \begin{aligned}&\Theta ^\textrm{max}_{[p_1,p_2]} = \left\{ x \in \Omega : \Theta _{[p_1,p_2]}(x) = \Vert \Theta _{[p_1,p_2]} \Vert _\infty \right\} , \end{aligned} \end{aligned}$$

and let $\mathcal {M}_j^\zeta$’s as well as $\mathcal {M}^\zeta$ denote the operator $\mathcal {M}_j$’s and $\mathcal {M}$ with $m_j$ replaced by $m_j^\zeta$. According to (Djidjou-Demasse et al. 2017, Theorem 2.2), for sufficiently small $\zeta$, the spectral radii satisfied:

$$ \begin{aligned}&r\left( \mathcal {M}_1^\zeta \right) =r\left( \mathcal {L}_1\right) = \Theta ^2_{[p_1,0]}(\bar{x}) + \mathcal {O}(\zeta ), \quad \text {for any } \bar{x} \in \Theta _{[p_1,0]}^\textrm{max}, \\&r\left( \mathcal {M}_2^\zeta \right) =r\left( \mathcal {L}_2\right) = \Theta ^2_{[0,p_2]}(\bar{x}) + \mathcal {O}(\zeta ), \quad \text {for any } \bar{x} \in \Theta _{[0,p_2]}^\textrm{max}, \\&r\left( \mathcal {M}^\zeta \right) = r\left( \mathcal {L}\right) =\Theta ^2_{[p_1,p_2]}(\bar{x}) + \mathcal {O}(\zeta ), \quad \text {for any } \bar{x} \in \Theta _{[p_1,p_2]}^\textrm{max}. \end{aligned} $$

#### Proof of Theorem 3.2

Finding steady states of Model (2.2)-(2.3) comes down to solving the following System:

3.14 $$\begin{aligned} {\left\{ \begin{array}{ll} \displaystyle h(u^*) \int _0^{\infty } \int _{\Omega } m(x,y) r(a,y) v^*(a,y)\textrm{d}a \textrm{d}y = \mathcal {N}(x) u^*(x),\\ v^*(0,x) =\mathcal {P} \mathcal {C}(x) u^*(x),\\ \partial _a v^*(a,x) = - \mathcal {D}(a,x)v^*(a,x), \end{array}\right. } \end{aligned}$$

with $$u^*(\cdot )=(E_{j}^*(\cdot ))_{j=0,1,2}$$ and $$v^*(\cdot ,\cdot )=(A_{j}^*(\cdot ,\cdot ))_{j=0,1,2}$$.

Solving (3.14) for $v^*$ yields,

3.15 $$\begin{aligned} v^*(a,x) = \boldsymbol{\Pi }(0,a,x)\mathcal {P} \mathcal {C}(x)u^*(x), \end{aligned}$$

where $\boldsymbol{\Pi }(0,a,x)$ is defined by (3.6).

The first equation of (3.14) combined with (3.15) leads to:

3.16 $$\begin{aligned} \int _{\Omega } m(x,y) \left( \int _0^\infty r(a,y)\boldsymbol{\Pi }(0,a,y) \textrm{d}a \right) \mathcal {P}\mathcal {C}(y)\mathcal {N}^{-1}(y){u}^{**}(y) \textrm{d}y = \frac{1}{h(u^*)} {u}^{**}(x), \end{aligned}$$

where $${u}^{**}=\mathcal {N}u^*$$, ie.,

$$ {u}^{**} =\textrm{diag} \left( \mu _{j}+\gamma _{j}\right) _{j=0,1,2} (E_{j}^*)_{j=0,1,2}.$$

Equality (3.16) rewrites as:

3.17 $$\begin{aligned} \mathcal {L}[{u}^{**}](x) = \frac{1}{h(u^*)}{u}^{**}(x), \end{aligned}$$

and then, the existence of $$u^{**}$$ is strongly linked to the spectral properties of the linear operator $$\mathcal {L}$$ defined by (3.11).

Let us set $$u^{**}=\left( u^{**}_0, u^{**}_1,u^{**}_2 \right) $$. We then consider three cases.

**Case 1:** $$\boldsymbol{p_1=0}$$ **and** $$\boldsymbol{p_2\in (0,1)}$$. By (3.14) and (3.10), we have $$u^{**}_1\equiv 0$$ (equivalently $$u^*_1=E^*_1\equiv 0$$) and

3.18 $$\begin{aligned} \mathcal {L}_2[(u^{**}_0,u^{**}_2)] = \zeta ^* (u^{**}_0,u^{**}_2), \end{aligned}$$

with

3.19 $$\begin{aligned} \zeta ^*= h(u^*)^{-1}, \end{aligned}$$

with the function *h* introduced by (3.2), and $\mathcal {L}_2$ the linear operator defined as

$$ \mathcal {L}_2: L_{+}^{1}(\Omega , \mathbb {R}^2) \ni \varphi \mapsto \mathcal {L}_2[\varphi ](\cdot )= \int _{\Omega } {m}(\cdot , y) \Gamma _2(y) \varphi (y)\textrm{d}y \in L_{+}^{1}(\Omega ,\mathbb {R}^2), $$

and

$$ \Gamma _2(y)= \textrm{diag} \left( 1 -p_{2}, p_2 \right) \left( \Gamma _{i,j}(y) \right) _{i,j\in \{0,2\}}. $$

As the linear operator $\mathcal {L}_2$ is positive, compact, and irreducible (see (Djidjou-Demasse et al. 2017, Theorem 4.1)), thanks to Krein-Rutman’s theorem, the spectral radius $\textrm{r}(\mathcal {L}_2)$ of $\mathcal {L}_2$ is positive, and there exists a function $\Psi _2=(\psi _2^0,\psi _2^2) \in L^1(\Omega ,\mathbb {R}^2)$, with $\Psi _2>0$ a.e., such that $\mathcal {L}_2[\Psi _2] = \textrm{r}(\mathcal {L}_2)\Psi _2$. Moreover, by (3.18), we have (see (Djidjou-Demasse et al. 2017, Theorem 4.1))

$$ \textrm{r}(\mathcal {L}_2) = \zeta ^*, \quad \text {and} \quad \textrm{diag} \left( \mu _{j}+\gamma _{j}\right) _{j=0,2} (E^*_0,E^*_2)= (u^{**}_0,u^{**}_2)= c \Psi _2, $$

with *c* a positive constant. From where, (3.15) gives

$$\begin{aligned}&A^*_1\equiv 0, \quad A^*_0(a,x) = c(1-p_2) \tau (x)\pi _0(0,a,x) \omega _2(x), \quad \\&A^*_2(a,x) = cp_2 \tau (x)\pi _2(0,a,x) \omega _2(x) , \end{aligned}$$

with

$$\omega _2= \frac{\gamma _0}{\mu _0+\gamma _0}\psi _2^0 + \frac{\gamma _2}{\mu _2+\gamma _2} \psi _2^2.$$

Therefore, by (3.19), it comes

$$ c= \left( \left( \textrm{r}(\mathcal {L}_2)\right) ^{1/\kappa }-1\right) C_\textrm{cte2}^{-1}, $$

with

3.20 $$\begin{aligned} C_\textrm{cte2}&= \sum _{j=0,2} \int _\Omega (\mu _j+\gamma _j)^{-1} \psi _2^j \textrm{d}x + (1-p_2) \int _\Omega \int _0^\infty \pi _0(0,\cdot ,\cdot ) \omega _2 \textrm{d}a \textrm{d}x \nonumber \\&\quad + p_2 \int _\Omega \int _0^\infty \pi _2(0,\cdot ,\cdot ) \omega _2 \textrm{d}a \textrm{d}x. \end{aligned}$$

Finally, we have

$$ \begin{aligned}&E_1^*\equiv 0, \quad A_1^*\equiv 0,\\&E_j^*= \left( \left( \textrm{r}(\mathcal {L}_2)\right) ^{1/\kappa }-1\right) C_\textrm{cte2}^{-1} (\mu _j+\gamma _j)^{-1}\psi _2^j, \quad j=0,2,\\&A^*_0(a,x) = (1-p_2) \left( \left( \textrm{r}(\mathcal {L}_2)\right) ^{1/\kappa }-1\right) C_\textrm{cte2}^{-1} \tau (x) \pi _0(0,a,x) \omega _2(x),\\&A^*_2(a,x) = p_2 \left( \left( \textrm{r}(\mathcal {L}_2)\right) ^{1/\kappa }-1\right) C_\textrm{cte2}^{-1} \tau (x) \pi _2(0,a,x) \omega _2(x). \end{aligned} $$

**Case 2:** $$\boldsymbol{p_1\in (0,1)}$$ **and** $$\boldsymbol{p_2=0}$$. By (3.14) and (3.10), we have $$u^{**}_2\equiv 0$$ (equivalently $$u^*_2=E^*_2\equiv 0$$) and

$$\begin{aligned} \mathcal {L}_1[(u^{**}_0,u^{**}_1)] = \xi ^* (u^{**}_0,u^{**}_1), \end{aligned}$$

with

$$\begin{aligned} \xi ^*= h(u^*)^{-1}, \end{aligned}$$

$$ \mathcal {L}_1: L_{+}^{1}(\Omega , \mathbb {R}^2) \ni \varphi \mapsto \mathcal {L}_1[\varphi ](\cdot )= \int _{\Omega } {m}(\cdot , y) \Gamma _1(y) \varphi (y)\textrm{d}y \in L_{+}^{1}(\Omega ,\mathbb {R}^2), $$

and

$$\Gamma _1(y)=\textrm{diag} \left( 1 -p_{1}, p_1 \right) \left( \Gamma _{i,j}(y) \right) _{i,j\in \{0,1\}}.$$

Applying the same reasoning as for case 1, we deduce that

$$ \begin{aligned}&E_2^*\equiv 0, \quad A_2^*\equiv 0,\\&E_j^*= \left( \left( \textrm{r}(\mathcal {L}_1)\right) ^{1/\kappa }-1\right) C_\textrm{cte1}^{-1} (\mu _j+\gamma _j)^{-1}\psi _1^j, \quad j=0,1,\\&A^*_0(a,x) = (1-p_1) \left( \left( \textrm{r}(\mathcal {L}_1)\right) ^{1/\kappa }-1\right) C_\textrm{cte1}^{-1} \tau (x) \pi _0(0,a,x) \omega _1(x),\\&A^*_1(a,x) = p_1 \left( \left( \textrm{r}(\mathcal {L}_1)\right) ^{1/\kappa }-1\right) C_\textrm{cte1}^{-1} \tau (x) \pi _1(0,a,x) \omega _1(x), \end{aligned} $$

with

$$\begin{aligned} \omega _1= \frac{\gamma _0}{\mu _0+\gamma _0}\psi _1^0 + \frac{\gamma _1}{\mu _1+\gamma _1} \psi _1^1, \end{aligned}$$

and

3.21 $$\begin{aligned} C_\textrm{cte1}&= \sum _{j=0}^1 \int _\Omega (\mu _j+\gamma _j)^{-1} \psi _1^j \textrm{d}x + (1-p_1) \int _\Omega \int _0^\infty \pi _0(0,\cdot ,\cdot ) \omega _1 \textrm{d}a \textrm{d}x \nonumber \\&\quad + p_1 \int _\Omega \int _0^\infty \pi _1(0,\cdot ,\cdot ) \omega _1 \textrm{d}a \textrm{d}x. \end{aligned}$$

**Case 3:** $\boldsymbol{p_1,p_2\in (0,1)}$. Thanks to (Djidjou-Demasse et al. 2017, Theorem 4.1) and Krein-Rutman’s theorem, the spectral radius $\textrm{r}(\mathcal {L})$ of $\mathcal {L}$ is positive, and there exists a function $\Psi =(\psi ^0, \psi ^1,\psi ^2) \in L^1(\Omega ,\mathbb {R}^3)$, with $\Psi >0$ a.e., such that $\mathcal {L}[\Psi ] = \textrm{r}(\mathcal {L})\Psi$. Moreover, by (3.17)

$$ \textrm{r}(\mathcal {L}) = \frac{1}{h(u^*)}, \quad \text {and} \quad u^* = c \mathcal {N}^{-1}\Psi , $$

with *c* a positive constant defined by,

$$ c= \left( \left( \textrm{r}(\mathcal {L})\right) ^{1/\kappa }-1\right) C_\textrm{cte}^{-1}, $$

where,

3.22 $$\begin{aligned} C_\textrm{cte} = \bar{h}(\mathcal {N}^{-1}\psi ) \end{aligned}$$

Finally,

$$ \begin{aligned}&E_j^*= \left( \left( \textrm{r}(\mathcal {L})\right) ^{1/\kappa }-1\right) C_\textrm{cte}^{-1} (\mu _j+\gamma _j)^{-1}\psi ^j, \quad j=0,1,2, \\&A^*_0(a,x) = (1-p_1-p_2) \left( \left( \textrm{r}(\mathcal {L})\right) ^{1/\kappa }-1\right) C_\textrm{cte}^{-1}\tau (x) \pi _0(0,a,x) \omega (x), \\&A^*_1(a,x) = p_1 \left( \left( \textrm{r}(\mathcal {L})\right) ^{1/\kappa }-1\right) C_\textrm{cte}^{-1}\tau (x) \pi _1(0,a,x) \omega (x),\\&A^*_2(a,x) = p_2 \left( \left( \textrm{r}(\mathcal {L})\right) ^{1/\kappa }-1\right) C_\textrm{cte}^{-1}\tau (x) \pi _2(0,a,x) \omega (x), \end{aligned} $$

with

$$\omega = \frac{\gamma _0}{\mu _0+\gamma _0}\psi ^0 + \frac{\gamma _1}{\mu _1+\gamma _1} \psi ^1 + \frac{\gamma _2}{\mu _2+\gamma _2} \psi ^2.$$

This concludes the proof of Theorem 3.2.

#### Proof of Theorem 3.3

Under assumption $\gamma _\ell = \gamma$ and $\mu _\ell = \mu$, observe that the operator $\mathcal {L}_2$ is then of the form: $$ \mathcal {L}_2=\begin{pmatrix} L_2 & L_2\\ \bar{L}_2 & \bar{L}_2 \end{pmatrix}, $$ with

$$ L_2\varphi = (1-p_2)\int _{\Omega } m_0(\cdot , y) \Gamma _{0,0}(y) \varphi (y)\textrm{d}y, \quad \bar{L}_2\varphi = p_2\int _{\Omega } m_2(\cdot , y) \Gamma _{2,2}(y) \varphi (y)\textrm{d}y. $$

To proceed further, we claim that (the proof will be provided later):

##### Claim 3.5

Let *X* be a Banach space, $M:X \rightarrow X$ and $N:X\rightarrow X$ two linear, positive, compact and irreducible operators. Set $$W= \begin{pmatrix} N & \quad N\\ M & \quad M \end{pmatrix}$$. Denote by $\textrm{r}(W)$ the spectral radius of *W* as well as $\psi$ the corresponding eigenfunction. Then,

$$ \textrm{r}(W)= \textrm{r}(M+N), \quad \text {and} \quad \psi = \left\| \begin{pmatrix} N \phi \\ M \phi \end{pmatrix} \right\| ^{-1} \begin{pmatrix} N \phi \\ M \phi \end{pmatrix}, $$

where $\phi >0$ is the eigenfunction associated to spectral radius $\textrm{r}(M+N)$ of $(M+N)$.

Consequently, by Claim 3.5, we have $\textrm{r}(\mathcal {L}_2)= \textrm{r}(L_2+ \bar{L}_2)$. Similar arguments apply for the cases of $\textrm{r}(\mathcal {L}_1)$ and $\textrm{r}(\mathcal {L})$.

We conclude the proof of Theorem 3.3 by providing the proof of Claim 3.5.

##### Proof of Claim 3.5

Since *W* is a linear positive, compact and irreducible operator, by the Krein-Rutman’s theorem, we can find $\psi =(u,v)>0$, with $\Vert \psi \Vert =1$, such that $W\psi = \textrm{r}(W)\psi$, with $\textrm{r}(W)>0$ the spectral radius of *W*, ie.,

3.23 $$\begin{aligned} \begin{pmatrix} N(u+v)\\ M(u+v) \end{pmatrix}= \textrm{r}(W) \begin{pmatrix} u\\ v \end{pmatrix}. \end{aligned}$$

Therefore, $$(N+M)(u+v)=\textrm{r}(W) (u+v)$$.

Since $N+M$ is a positive, compact and irreducible operator, by the Krein-Rutman’s theorem, we have $\textrm{r}(W)= \textrm{r}(M+N)$ and $u+v =c_0 \phi$, where $c_0$ is a positive constant and $\phi$ is the eigenvector associated the spectral radius $\textrm{r}(M+N)$ of $N+M$.

By (3.23), we have

$$ u= \frac{c_0 }{\textrm{r}(M+N)} N \phi , \quad v= \frac{c_0 }{\textrm{r}(M+N)} M \phi . $$

As $\Vert \psi \Vert =\Vert (u,v)\Vert =1$, it comes

$$ u= \left\| \begin{pmatrix} N \phi \\ M \phi \end{pmatrix} \right\| ^{-1} N \phi , \quad v= \left\| \begin{pmatrix} N \phi \\ M \phi \end{pmatrix} \right\| ^{-1} M \phi . $$

This ends the proof of Claim 3.5. $\square$

## Model parameterization and quantitative variable

The values and ranges of all constant parameters are provided in Table 1. Other functional parameters, such as the proportion of eggs laid $\gamma _j$’s and the rate at which eggs become unviable $\mu _j$’s, are also assumed to be constant, with their values specified in Table 1.

**The mutation kernel.** The mutation probability $m_j$ is defined as

4.1 $$\begin{aligned} m_j(x,y)= \mathcal {G}(0, \mathrm{var_j})(x-y), \end{aligned}$$

where $\mathcal {G}(0, \mathrm{var_j})$ stands for the normal probability density distribution with mean 0 and standard deviation $\mathrm{var_j}$. The mutational variance $\textrm{var}_0$ within the unexposed mosquito population is considered variable, with values provided in Table 1. For mosquito populations in insecticide state $j \in \{1,2\}$, the mutational variance $\textrm{var}_j$ is defined as $\textrm{var}_j = \textrm{var} \times \textrm{var}_0$, where the mutational variance ratio $\textrm{var}$ is also assumed to be variable, with its values specified in Table 1. Moreover, the Gaussian kernel is used here only as an illustrative example; other kernels may be considered as long as Assumption 2.2 is satisfied.

**Egg-laying rate.** Using a similar framework as in Djidjou-Demasse et al. (2023), the egg-laying rate $r(a,x)$ of an AFM (adult female mosquito) of age $a$ and resistance level $x$ is defined by:

4.2 $$\begin{aligned} r(a,x)=r_m \left[ 1+\left( \frac{r_m-r_0}{r_0}\right) \left( \frac{r_0}{r_1}\cdot \frac{r_m-r_1}{r_m-r_0} \right) ^x \right] ^{-1} \times \textbf{1}_{a>a_\textrm{L}}, \end{aligned}$$

where $r_m$ is a constant due to physiological constraints and $a_\textrm{L}$ is the average age at which AFMs start laying eggs. The constants $r_{0}$ and $r_{1}$ are egg-laying rates of the reference ’sensitive’ and ’resistant’ mosquitoes to insecticide with $0<r_{1}<r_{0}<r_{m}$. The constants $r_i$’s are given in Table 1.

**Adult mosquitoes death rate.** The death rate $d_{0}(a,x)$ of unexposed adult mosquitoes aged *a* with resistant level *x* is such that

4.3 $$\begin{aligned} d_{0}(a,x)= \kappa _0 \textbf{1}_{a>a_\textrm{LS}}, \end{aligned}$$

where $a_\textrm{LS}$ is the average lifespan of mosquitoes, and $\kappa _0$ is a positive constant. Note that the average lifespan of unexposed mosquitoes is given by $\int _0^\infty \pi _0(0,a,x) \textrm{d}a= a_\textrm{LS} + \frac{1}{\kappa _0}$. Since $a_\textrm{LS}$ is fixed around 21 days, therefore, as soon as the constant $\kappa _0$ is sufficiently large, e.g., $\kappa _0=10$, we have $\int _0^\infty \pi _0(0,a,x) \textrm{d}a= a_\textrm{LS} + \frac{1}{\kappa _0} \approx a_\textrm{LS}.$

For adult mosquitoes in the *j*-insecticide state $(j=1,2)$, their death rate $d_{j}(a,x)$ accounts for the insecticide pressure such that

4.4 $$\begin{aligned} d_{j}(a,x)= d_{0}(a,x) + \bar{d}_{0,j} \left( \dfrac{\bar{d}_{j}}{\bar{d}_{0,j}} \right) ^x, \end{aligned}$$

where $d_0(a,x)$ represents the baseline mortality rate in the absence of insecticide exposure.

The second term models the additional mortality induced by insecticide $\textrm{I}_{j}$. Here, $\bar{d}_{0,j}$ and $\bar{d}_j$ correspond respectively to the insecticide-induced death rates of a reference sensitive mosquito (labeled as resistance level $x=0$) and a reference resistant mosquito (labeled resistance level $x=1$). The expression $\bar{d}_{0,j} \left( \frac{\bar{d}_j}{\bar{d}_{0,j} } \right) ^x$ provides an exponential interpolation between these two extremes, allowing the insecticide-induced mortality to decrease smoothly and nonlinearly as resistance increases.

**Fig. 1:**
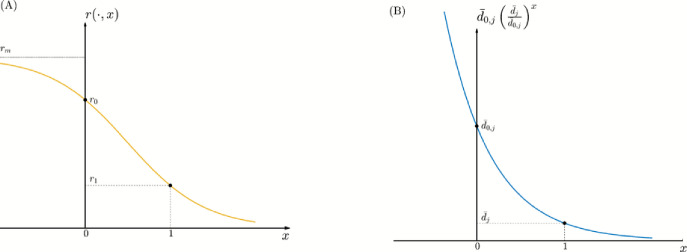
(A) Eggs-laying rate, $r(\cdot ,x)$, of AFMs with resistance level *x*. (B) Insecticide activity, $\bar{d}_{0,j} \left( \frac{\bar{d}_j}{\bar{d}_{0,j}} \right) ^x$, on AFMs population with resistance level *x*

Finally, note that, in general, the function $r$ is assumed to be bounded (Assumption 2.1), which ensures control of egg-laying rate as a function of the resistance phenotype $x$. Moreover, the qualitative shapes of the functions $r(\cdot , x)$ and $\bar{d}_{0,j} \left( \frac{\bar{d}_j}{\bar{d}_{0,j} } \right) ^x$ are shown in Figure 1. Importantly, the specific functional forms of $r$ and $d_j$ are not required for the formulation or analysis of the model; the essential assumption is that both functions monotonically decrease with respect to the resistance level $x$, capturing the fitness cost of resistance through the function $r$ and the fitness advantage of resistance through the function $d_j$.

**Initial conditions before deployment of insecticide** $\textrm{I}_{2}$. We assume that, after a prolonged period of exclusive use of insecticide $\textrm{I}_{1}$, with an exposure rate denoted by $p_0$, the mosquito population reaches a stationary state. The parameter $p_0$ represents the overall initial insecticide pressure in the environment.

**Fig. 2:**
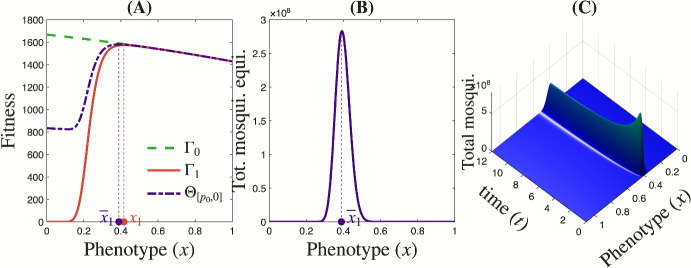
**Evolutionary dynamics under exclusive use of insecticide** $\textrm{I}_{1}$ **at a proportion** $p_0 = 0.5$. **(A)** The fitness functions. **(B)** The total mosquito population at evolutionary equilibrium is largely concentrated around the dominant phenotype $\bar{x}_1$, which maximizes the fitness function $\Theta _{[p_0,0]}$, in the insecticide landscape composed only of $\textrm{I}_{1}$ in proportion $p_1=p_0$. **(C)** Dynamics of total mosquito population size and distribution across phenotypic values.

At time $t = 0$, insecticide $\textrm{I}_{2}$ is introduced into the insecticide landscape. At the stationary state prior to the introduction of $\textrm{I}_{2}$, the fitness function $\Theta _{[p_0,0]}$, defined in (3.12), is maximized at the phenotypic value $\bar{x}_1 \in \Theta _{[p_0,0]}^\textrm{max}$ within a landscape composed solely of insecticide $\textrm{I}_{1}$ at proportion $p_1 = p_0$ (Figure 2A), with $\Theta _{[p_0,0]}^\textrm{max}$ defined in (3.13). At evolutionary equilibrium, the mosquito population concentrates around this dominant phenotypic value (Figures 2B,C).

The new insecticide $\textrm{I}_{2}$ is characterized such that the fitness $\Gamma _2(x_1)$ of mosquitoes with resistance level $x_1$ under its selective pressure satisfies

**Fig. 3:**
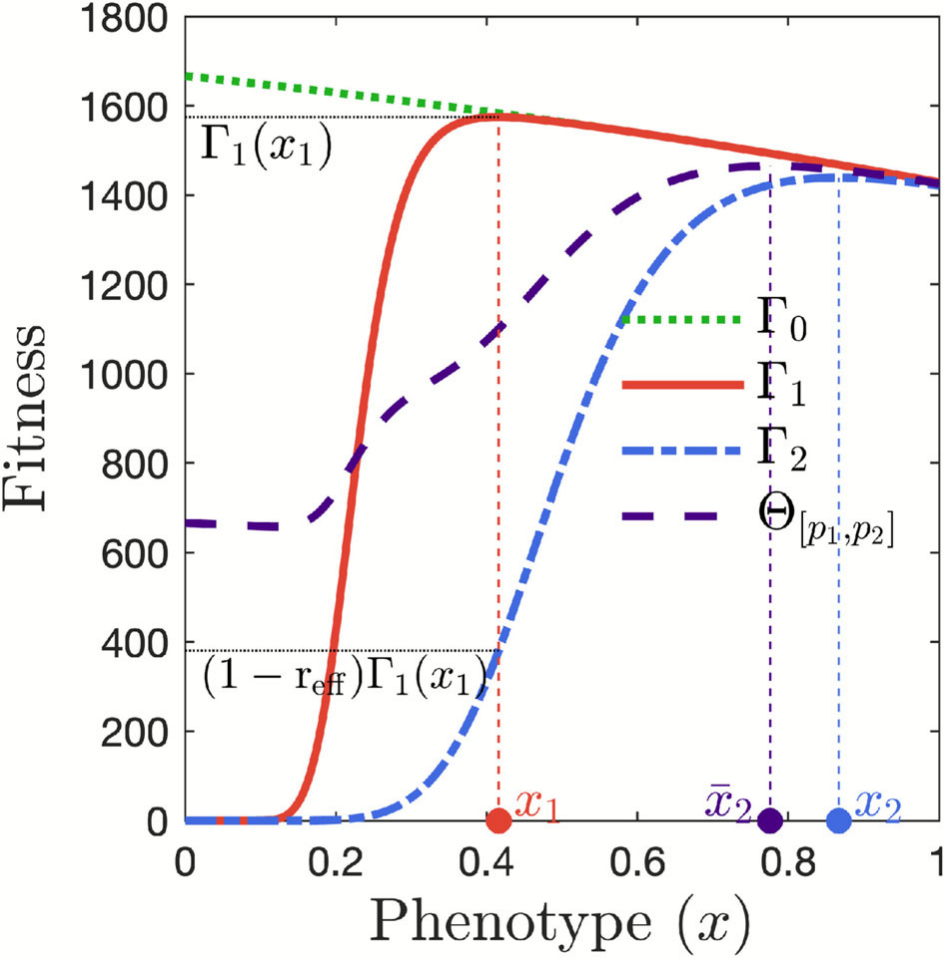
**The fitness landscape.** Prior to the introduction of insecticide $\textrm{I}_{2}$, the dominant mosquito phenotype is denoted by $x_1$. The relative effectiveness parameter $\mathrm{r_{eff}}$ measures the fitness reduction of phenotype $x_1$ under $\textrm{I}_{2}$ compared to $\textrm{I}_{1}$, and is defined such that $\Gamma _2(x_1) = (1 - \mathrm{r_{eff}}) \Gamma _1(x_1)$. When insecticide $\textrm{I}_{2}$ is used alone, the dominant phenotype shifts to $x_2$. In a mixed insecticide landscape with proportions $p_1$ and $p_2$ of $\textrm{I}_{1}$ and $\textrm{I}_{2}$, respectively, the fitness function $\Theta _{[p_1,p_2]}$ reaches its maximum at a new dominant phenotype, denoted by $\bar{x}_2$. In the scenario illustrated here, $p_0 = 0.5$, $p_1=0.2$, $p_2=0.4$, and $\mathrm{r_{eff}} = 0.75$.

4.5 $$\begin{aligned} \Gamma _2(x_1) = (1 - \mathrm{r_{eff}} ) \, \Gamma _1(x_1), \end{aligned}$$

where $x_1$ is the optimal phenotype with respect to the fitness function $\Gamma _1$. The parameter $\mathrm{r_{eff}} \in (0,1)$ quantifies the relative effectiveness of insecticide $\textrm{I}_{2}$ compared to $\textrm{I}_{1}$ against the initial dominant phenotype established at evolutionary equilibrium under exclusive use of $\textrm{I}_{1}$ (Figure 3).

Let $p_1^S \in (0,1)$ be given and fixed, denoting the probability of surviving $\textrm{I}_{1}$-insecticide exposure during one day. We set $\bar{d}_{0,1}= -\log \left( p_1^S \right)$ and $\bar{d}_1= -\log \left( 1-p_1^S \right)$. From here, the fitness function $\Gamma _1$ defined by (3.10) is fully parameterized. For the parameterization of the fitness function $\Gamma _2$, the probability $p_2^S$ of surviving $\textrm{I}_{2}$-insecticide exposure during one day is then determined such that (4.5) holds, i.e., for a given value of $\mathrm{r_{eff}} \in (0,1)$, $p_2^S$ satisfies

4.6 $$\begin{aligned} \int _0^\infty r(a,x_1) e^{-\int _0^a d_0(\sigma ,x_1) \textrm{d}\sigma } \exp \left( a \cdot \log \left( p_2^S \right) \left( \dfrac{\log \left( 1-p_2^S \right) }{\log \left( p_2^S \right) } \right) ^{x_1} \right) \textrm{d}a = \left( 1- \mathrm{r_{eff}} \right) \vartheta _1 , \end{aligned}$$

with $\vartheta _1= \Gamma _1(x_1) \left( \frac{\tau (x_1)\gamma _{2}(x_1)}{\mu _{2}(x_1) + \gamma _{2}(x_1)} \right) ^{-1}.$ While (4.6) can be solved numerically, obtaining an explicit expression for $p_2^S$ from (4.6) is generally not straightforward.

**Deployment strategies.** Insecticide $$\textrm{I}_{2}$$ may be deployed in combination with insecticide $$\textrm{I}_{1}$$ during the $$\overline{T}$$ years insecticide implementation period. The composition of the insecticide landscape is updated *N* times at regular intervals, occurring at times $$T_0$$, $$T_1$$, $$\cdots $$, $$T_{N-1}$$ introduced previously. During each interval $$[T_{n-1},T_n)$$, with $$n\in \{1,\cdots , N\}$$, the landscape is characterized by proportions $$p^n_1$$ and $$p^n_2$$, corresponding to the proportions of insecticides $$\textrm{I}_{1}$$ and $$\textrm{I}_{2}$$, respectively, with $$p^n_1 + p^n_2 \le 1$$. A deployment strategy, denoted as $$\textrm{D}_\textrm{ep}\,\textrm{S}$$, over a period of $$\overline{T}$$ years is defined by the temporal composition of the insecticide landscape, given by:

$$ \textrm{D}_\textrm{ep}\textrm{S} = \begin{pmatrix} p_1^1& p_1^2 & \cdots & p_1^N\\ p_2^1& p_2^2 & \cdots & p_2^N \end{pmatrix}. $$

In the sequel, we set $p_j= \left( p_j^1, p_j^2, \cdots , p_j^N \right)$ to denote the corresponding proportions of insecticides $\textrm{I}_{j}$, with $j=1,2$.

We consider three deployment strategies: (i) a simple rotation strategy (denoted Rot), which alternates the use of insecticides $\textrm{I}_{1}$ and $\textrm{I}_{2}$ in successive update periods; (ii) a simple mosaic strategy (denoted Mos), in which each update period consists of an equal split of the insecticide landscape – 50% $\textrm{I}_{1}$ and 50% $\textrm{I}_{2}$; and (iii) an optimal mosaic strategy (denoted Mos*), which identifies the best combination of $\textrm{I}_{1}$ and $\textrm{I}_{2}$ proportions across update periods to minimize the mosquito population size over the entire deployment period.

**Relative gain.** The relative gain, $\textrm{R}_\textrm{gain} = \textrm{R}_\textrm{gain}\left( \textrm{D}_\textrm{ep}\textrm{S},p_0\right)$, from using insecticide $\textrm{I}_{2}$ over a period of $\overline{T}$ years is calculated as the relative reduction in mosquito population size compared to the baseline population size observed under continuous usage of insecticide $\textrm{I}_{1}$ alone with exposure rate $p_0$. Formally, this is defined as:

$$ \textrm{R}_\textrm{gain}\left( \textrm{D}_\textrm{ep}\textrm{S},p_0\right) = 1 - \frac{ \left. \sum _{j=0}^2 \int _{0}^{\overline{T}} \int _\Omega \int _0^\infty A_j(t,a,x) \textrm{d}a \textrm{d}x \textrm{d}t \right| _{\textrm{D}_\textrm{ep}\textrm{S}} }{\overline{T} \left. \int _\Omega \int _0^\infty \left( \bar{A}_0(a,x)+ \bar{A}_1(a,x) \right) \textrm{d}a \textrm{d}x \right| _{p_0} }, $$

where the numerator of the fraction denotes the size of the mosquito population during the $\overline{T}$ years deployment of insecticide $\textrm{I}_{2}$ with landscape composition strategy $\textrm{D}_\textrm{ep}\textrm{S}$, and the denominator denotes the mosquitoes population size at the stationary state under the baseline usage of $\textrm{I}_{1}$ only at the constant exposure rate $p_0$. For example, a relative gain $\textrm{R}_\textrm{gain}\left( \textrm{D}_\textrm{ep}\textrm{S},p_0\right)$ of 5% indicates that strategy *p* reduces the mosquito population size over the $\overline{T}$ years deployment period by 5%, whereas a negative value implies an increase in population size compared to the reference strategy where only $\textrm{I}_{1}$ is used at a constant exposure rate $p_0$.

**Time to resistance emergence.** The evolutionary output considered is the emergence time, denoted $\textrm{T}_\textrm{emerg}$, of the adapted strain $x_2$, ie., the resistance level that maximizes the fitness function $\Gamma _2$ under the selective pressure of insecticide $\textrm{I}_{2}$. Formally, $\textrm{T}_\textrm{emerg}$ is defined as: (i) the first time at which the proportion of mosquitoes with resistance level $x_{2}$ (denoted as $Q_{x_{2}}$) exceeds $10\%$ of the total population, and (ii) remains persistently higher than that of the previously dominant strain $\bar{x}_{1}$. More precisely, $\textrm{T}_\textrm{emerg}$ is the time from which, for all $t\ge \textrm{T}_\textrm{emerg}$, we have:

$$ Q_{x_2}(t):= \frac{ \sum _{j=0}^2 \int _0^\infty A_j(t,a,x_2) \textrm{d}a }{ \sum _{j=0}^2 \int _0^\infty \left( A_j(t,a,\bar{x}_0) + A_j(t,a,x_2) \right) \textrm{d}a }> 10\%. $$

The first condition (i) signals the initial emergence of strain $x_{2}$ from rarity to a significant frequency threshold. This threshold is chosen as a practical indicator that strain $x_{2}$ is no longer negligible in the population. The second condition (ii) ensures that this strain $x_{2}$ is not only established but that its increase is sustained over time.

## Results

### Typical dynamics simulated with the model

**Fig. 4:**
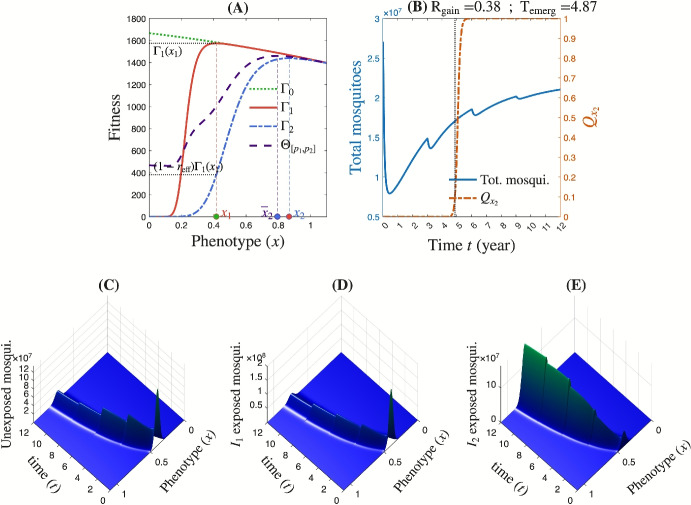
**Evolutionary dynamics with a deployment strategy** $p_1 = (0.24, 0.2, 0.16, 0.1)$, $p_2 = (0.48, 0.6, 0.72, 0.8)$. Here, we have $p_0 = 0.6$, $\mathrm{r_{eff}} = 0.75$, and $\textrm{var}_0=0.002$. **(A)** The fitness functions. **(B)** Dynamics of total population and population with phenotypic value $x_2$. The vertical dot line represents the time of resistance emergence $\textrm{T}_\textrm{emerg}$. **(C-E)** The evolutionary dynamics for unexposed and exposed adult mosquitoes

Introducing insecticide $\textrm{I}_{2}$ into a landscape initially composed solely of insecticide $\textrm{I}_{1}$ at a proportion $p_0 = 0.6$ results in a mixed insecticide environment with $\textrm{I}_{1}$ and $\textrm{I}_{2}$. The proportions of $\textrm{I}_{1}$ and $\textrm{I}_{2}$ are updated at regular intervals of duration $\mathrm{D_{ur}} = 3$ years over a total deployment period of $\overline{T} = 12$ years. We consider a deployment strategy consisting of: $p_1 = (0.24, 0.2, 0.16, 0.1)$ and $p_2 = (0.48, 0.6, 0.72, 0.8)$, corresponding to the proportions of $\textrm{I}_{1}$ and $\textrm{I}_{2}$, respectively, during each update period. In this new context (at least during the first three years), the fitness function $\Theta _{[p_1,p_2]}$ attains its maximum at a dominant phenotype denoted by $\bar{x}_2$ (Figure 4A). Under this deployment scenario, the relative gain achieved by introducing $\textrm{I}_{2}$ is approximately $\textrm{R}_\textrm{gain}\left( \textrm{D}_\textrm{ep}\textrm{S},p_0\right) \approx 38\%$, while the time to resistance emergence is estimated at $\textrm{T}_\textrm{emerg} \approx 4.9$ years (Figure 4B). These quantities are captured through the total mosquito population size and the proportion of mosquitoes with the phenotypic value $x_2$, which maximizes the fitness function $\Gamma _2$ under the selective pressure of insecticide $\textrm{I}_{2}$ (Figure 4B). The model captures the evolutionary dynamics of the mosquito population following the introduction of the insecticide regimen by simultaneously tracking changes in population size and resistance trait distribution (Figures 4C–E). Initially, i.e., during the first cycle corresponding to the first 3 years of deployment of insecticide $\textrm{I}_{2}$ at a proportion of 0.48, the regimen induces a sharp decline in the mosquito population size, followed by a great rebound in this cycle (Figure 4B). In the second and third cycles, increasing the proportion of $\textrm{I}_{2}$ to 0.6 and 0.72, respectively, results in a slight decline in mosquito population size over a relatively short period, followed by a rapid rebound (Figure 4B). In the final cycle, increasing the proportion of $\textrm{I}_{2}$ to 0.8 leads to a slight and temporary decline in mosquito population size. However, the subpopulation carrying the phenotypic trait $x_2$, associated with resistance to insecticide $\textrm{I}_{2}$, begins to expand one year after the beginning of the second cycle. Ultimately, as this resistant phenotype becomes dominant, the mosquito population escapes control under the current composition of the insecticide landscape, i.e., under the current deployment strategy $[p_{1},p_{2}]$ (Figure 4B).

### Sensitivity analysis

We assess the sensitivity of the relative gain ($\textrm{R}_\textrm{gain}$) and the time to resistance emergence ($\textrm{T}_\textrm{emerg}$) to five key parameters: the insecticide exposure rate prior to the deployment strategy ($p_0$), the insecticide landscape composition defined by the proportion $p_1$ of insecticide $\textrm{I}_{1}$, the relative effectiveness of insecticide $\textrm{I}_{2}$ compared to $\textrm{I}_{1}$ ($\mathrm{r_{eff}}$), the mutational variance in the unexposed adult population ($\textrm{var}_0$), and the mutational variance ratio ($\textrm{var}$). The parameter ranges explored are listed in Table 1.

**Fig. 5:**
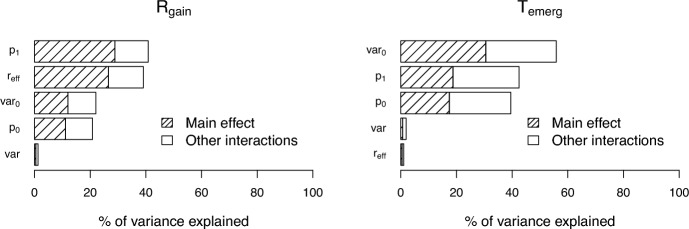
**Global sensitivity analysis.** Sensitivity of the relative gain ($\textrm{R}_\textrm{gain}$) and the time to resistance emergence ($\textrm{T}_\textrm{emerg}$) to key parameters: initial insecticide $\textrm{I}_{1}$ exposure rate before the deployment of $\textrm{I}_{2}$ ($p_0$), landscape composition defined by the proportion $p_1$ of insecticide $\textrm{I}_{1}$, relative effectiveness of $\textrm{I}_{2}$ vs. $\textrm{I}_{1}$ ($\mathrm{r_{eff}}$), mutational variance in unexposed AFM populations ($\textrm{var}_0$), and the mutational variance ratio ($\textrm{var}$). The hatched portions of the bars represent the main indices (the effect of each factor alone), while the complete bars, including both the hatched and unshaded parts, represent the total effect of each factor, accounting for interactions with all other factors

Sensitivity analysis identified the insecticide landscape composition and the relative effectiveness of insecticide $\textrm{I}_{2}$ compared to $\textrm{I}_{1}$ as the primary drivers of the relative gain $\textrm{R}_\textrm{gain}$, accounting for 29% and 27% of its variance, respectively (Figure 5). These are followed by the mutational variance in the unexposed AFM population (12%) and the insecticide exposure rate (11%). In contrast, the main determinant of the time to resistance emergence $\textrm{T}_\textrm{emerg}$ is the mutational variance in the unexposed AFM population, explaining 31% of its variance, followed by the insecticide landscape composition (19%) and the insecticide exposure rate (17%). The mutational variance ratio has a negligible influence on both $\textrm{R}_\textrm{gain}$ and $\textrm{T}_\textrm{emerg}$, contributing less than 0.4% to the variation in either outcome. Consequently, a potential mutagenesis component–captured by a possible mutational difference between unexposed and insecticide-exposed mosquitoes–plays only a marginal overall role.

While the relative effectiveness of insecticide $\textrm{I}_{2}$ compared to $\textrm{I}_{1}$ ($\mathrm{r_{eff}}$) accounts for 27% of the variation in the relative gain $\textrm{R}_\textrm{gain}$, it explains only 0.4% of the variation in the emergence time $\textrm{T}_\textrm{emerg}$ (Figure 5). This result highlights a trade-off between achieving a significant reduction in mosquito population size through insecticide pressure and ensuring the long-term sustainability of insecticide effectiveness by limiting the pace of resistance emergence within mosquito populations.

### Optimal deployment strategies

**Fig. 6:**
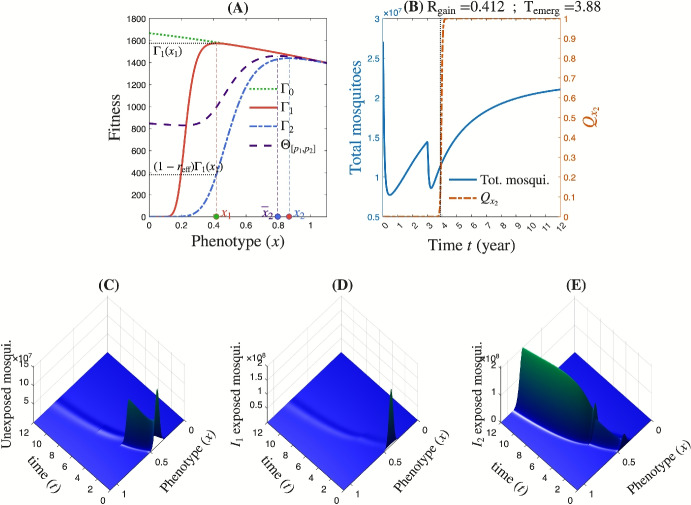
**Evolutionary dynamics with the optimal deployment strategy for a prior exposure rate of** $p_0 = 0.6$, $\mathrm{r{eff}} = 0.75$, **and** $\textrm{var}_0=0.002$. We established that the optimal mosaic strategy is obtained for $p_1 \approx (0.0023, 0.0024, 0.0027, 0.0031)$ and $p_2 \approx (0.4898, 0.9931, 0.9943, 0.9938)$. **(A)** the fitness functions. **(B)** Dynamics of total population and population with phenotypic value $x_2$. The vertical dot line represents the time of resistance emergence $\textrm{T}_\textrm{emerg}$. **(C-E)** The evolutionary dynamics for unexposed and exposed adult mosquitoes

We determined the optimal deployment strategy (Mos*) through a constrained nonlinear multivariable optimization, implemented with the $\mathrm fmincon$ function in MATLAB (version 2023a). Under the same parameter configuration as in Figure 4, the optimal solution prescribes proportions of $\textrm{I}_{1}$ and $\textrm{I}_{2}$ over successive 3-year update periods as $p_1 \approx (0.0023, 0.0024, 0.0027, 0.0031)$ and $p_2 \approx (0.4898, 0.9931, 0.9943, 0.9938)$, respectively. This outcome indicates that the Mos* strategy essentially phases out the inefficient insecticide $\textrm{I}_{1}$ while progressively introducing the more effective insecticide $\textrm{I}_{2}$. Implementing this strategy yields a relative gain of approximately $\textrm{R}_\textrm{gain}\left( \textrm{D}_\textrm{ep}\textrm{S},p_0\right) \approx 41.2\%$ (Figure 6B), corresponding approximately to a two-fifths reduction in mosquito population size. The time to resistance emergence is estimated at $\textrm{Temerg} \approx 3.9$ years (Figure 6B).

The evolutionary dynamics reveal that during the first 3 years of $\textrm{I}_{2}$ deployment, mosquito density undergoes a sharp decline followed by a rapid rebound (Figure 6B). In the second cycle, when the proportion of $\textrm{I}_{2}$ is increased to 99%, a slight temporary decrease in mosquito population size is again observed, quickly followed by resurgence. During the third and fourth cycles, maintaining $\textrm{I}_{2}$ at 99% results in sustained population growth. This pattern arises because the subpopulation carrying trait $x_2$, associated with resistance to $\textrm{I}_{2}$, begins to expand the first year of the second cycle and becomes dominant thereafter, ultimately driving the failure of the vector control strategy under the current insecticide landscape (Figure 6B–E).

**Fig. 7:**
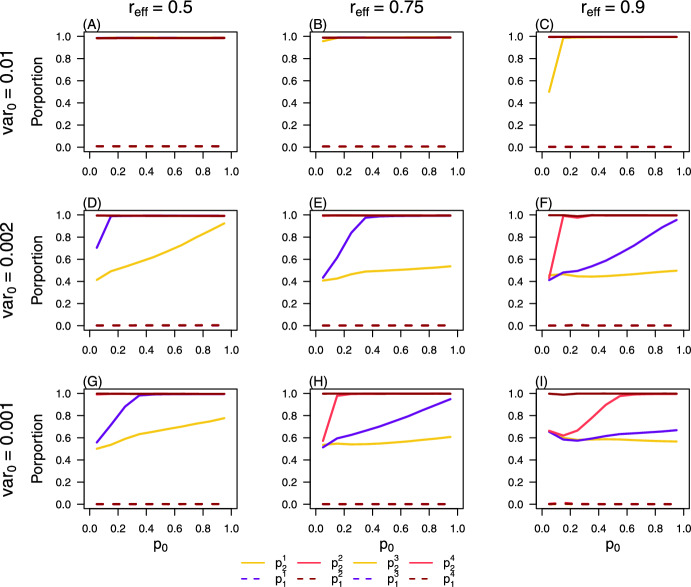
**The insecticide landscape with the mosaic optimal strategy.** The mutational variance ($\textrm{var}_0$) and the relative effectiveness ($\mathrm{r{eff}}$) of insecticide $\textrm{I}_{2}$ compared to $\textrm{I}_{1}$ are variables. With $n \in \{1, \cdots , 4\}$, $p_1^n$ and $p_2^n$ correspond to the proportions of insecticides $\textrm{I}_{1}$ and $\textrm{I}_{2}$ during the *n*-th updating period

Overall, the optimal mosaic strategy (Mos*) fully excluded insecticide $\textrm{I}_{1}$ regardless of the mutational variance within unexposed mosquito populations ($\textrm{var}_0$), the relative efficacy of $\textrm{I}_{2}$ compared to $\textrm{I}_{1}$ ($\mathrm{r{eff}}$) or the initial exposure rate to $\textrm{I}_{1}$ ($p_0$) prior to $\textrm{I}_{2}$ deployment (Figure 7). This means that, under the hypothesis that the first insecticide is ineffective against mosquitoes, simultaneous use of both insecticides is rarely optimal.

For high mutational variance within unexposed mosquito populations, the optimal mosaic strategy (Mos*) consists exclusively of insecticide $\textrm{I}_{2}$ at full (100%) coverage, regardless of the values of $\mathrm{r{eff}}$ or $p_0$ (Figure 7A–C). By contrast, when $\textrm{var}_0$ is relatively small (Figure 7D–F), insecticide $\textrm{I}_{1}$ is fully excluded from the optimal strategy. However, the proportion of $\textrm{I}_{2}$ during the first deployment period is only moderate, except when $\mathrm{r{eff}}$ is low and $p_0$ is high. During the second deployment period, the proportion of $\textrm{I}_{2}$ rapidly approaches 100% once $p_0$ exceeds a certain threshold, and from the third period onward, $\textrm{I}_{2}$ is maintained at nearly 100% coverage for all values of $p_0$. Finally, for very small values of $\textrm{var}_0$ (Figure 7G–I), the qualitative pattern is similar to that observed in panels D–F. However, the threshold value of $p_0$ at which $\textrm{I}_{2}$ reaches full deployment in the second period is shifted to higher values. Moreover, in this regime, the proportion of $\textrm{I}_{2}$ during the third deployment period does not consistently remain close to 100%, particularly when $\mathrm{r{eff}}$ is high and $p_0$ is moderate (Figure 7H–I).

### Influence of key parameters on the effectiveness of deployment strategies

**Fig. 8:**
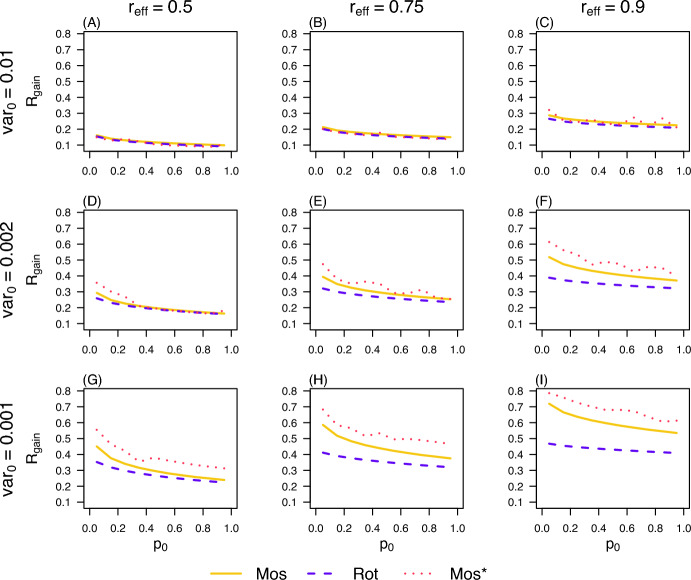
The relative gain with strategies Rot, Mos, and Mos*. The mutational variance ($\textrm{var}_0$) and the relative effectiveness ($\mathrm{r{eff}}$) of insecticide $\textrm{I}_{2}$ compared to $\textrm{I}_{1}$ are variables

Regardless of the deployment strategy– whether the simple mosaic (Mos), the simple rotation (Rot), or the optimal mosaic (Mos*)– the mutational variance ($\textrm{var}0$) and the relative effectiveness of insecticide $\textrm{I}_{2}$ compared to $\textrm{I}_{1}$ ($\mathrm{r{eff}}$) have consistent effects on outcomes: the relative gain $\textrm{R}_\textrm{gain}$ systematically decreases as the initial insecticide exposure rate ($p_0$), prior to the introduction of $\textrm{I}_{2}$, increases (Figure 8). This highlights that strong insecticide pressure before the deployment phase diminishes the achievable gains in mosquito population suppression. Furthermore, the Mos strategy consistently outperforms Rot, particularly when $\textrm{var}0$ is low and $\mathrm{r{eff}}$ is high (Figure 8G–I). This result is intuitive, as the Rot strategy requires periodically reintroducing the already inefficient insecticide $\textrm{I}_{1}$ at 100% coverage during certain update periods, an approach that is inherently inconsistent with maintaining effective population suppression. Finally, the Mos* strategy provides a marked performance advantage over the simple Mos strategy when $\textrm{var}0$ is low, and this advantage remains largely robust to variations in $\mathrm{r{eff}}$ (Figure 8G–I).

## Conclusion and discussion

The extensive use of pyrethroid-based insecticides has led to the emergence and proliferation of insecticide resistance (IR) in mosquito populations, posing a significant threat to malaria control and elimination efforts, which rely heavily on vector control through insecticide interventions. Given the limited arsenal of insecticides available for public health applications, there is an urgent need to develop and implement effective insecticide resistance management (IRM) strategies to ensure the sustainable use of these compounds. Building upon previous research (Kezeta-Bondja et al. 2025), we model insecticide resistance as a quantitative trait, reflecting its polygenic nature rather than a monogenic basis. This approach enabled a more accurate representation of the transient evolutionary dynamics associated with resistance development. We integrate this trait-based resistance model into an age-structured framework governed by integro-differential equations, wherein the continuous resistance trait modulates key life-history parameters, including oviposition and mortality. We assess the efficacy of three insecticides deployment strategies –simple mosaic (Mos), simple rotation (Rot), and optimal mosaic (Mos*)– in their ability of reducing mosquito population size, thereby contributing novel insights to the existing body of literature on IRM.

The model’s mathematical properties were rigorously analyzed, including the existence of a unique maximal bounded semiflow and conditions for the existence of steady states. Through parameterization and numerical simulations, we explored both epidemiological outcomes– quantified by the relative gain of introducing insecticide $\textrm{I}_{2}$ into the insecticides landscape– and evolutionary dynamics, characterized by the time of resistance emergence. Comparative analysis of the three strategies revealed their differential impacts on mosquito population reduction following the deployment of $\textrm{I}_{2}$. These findings offer valuable guidance for the design of sustainable and adaptive vector control strategies.

**Fig. 9:**
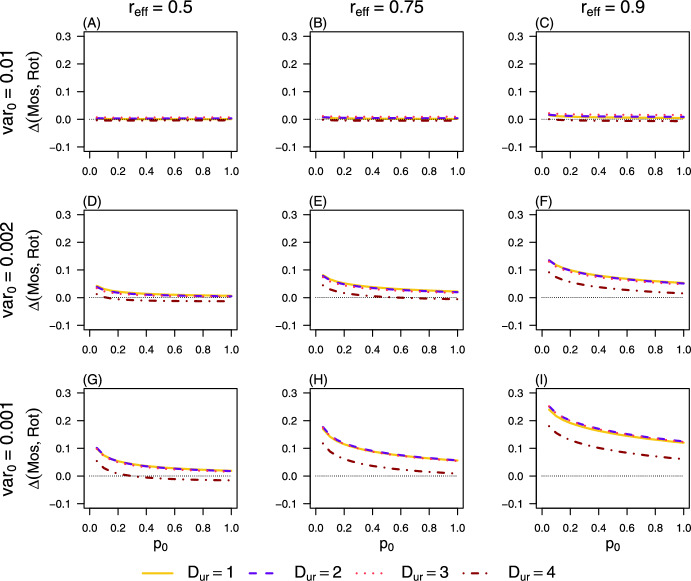
**The difference in performance between Mos and Rot strategies.** Here $\Delta (\text {Mos},\text {Rot}) = \textrm{R}_\textrm{gain}(\text {Mos}) - \textrm{R}_\textrm{gain}(\text {Rot})$. The mutational variance ($\textrm{var}_0$) and the relative effectiveness ($\mathrm{r{eff}}$) of insecticide $\textrm{I}_{2}$ compared to $\textrm{I}_{1}$ are variables.

While the Mos consistently outperforms the Rot strategy, regardless of the mutational variance ($\textrm{var}_0$) and the relative effectiveness of insecticide $\textrm{I}_{2}$ compared to $\textrm{I}_{1}$ ($\mathrm{r_{eff}}$) (Figure 8), the difference in performance between Mos and Rot strategies – denoted by $\Delta (\text {Mos},\text {Rot}) = \textrm{R}_\textrm{gain}(\text {Mos}) - \textrm{R}_\textrm{gain}(\text {Rot})$ – is influenced by the insecticide landscape updating interval ($\mathrm{D_{ur}}$). Specifically, values of $\mathrm{D_{ur}}$ ranging from 1 to 3 years have a negligible impact on the performance difference $\Delta (\text {Mos},\text {Rot})$. In contrast, when $\mathrm{D_{ur}} = 4$ years, the performance advantage of the Mos strategy over Rot is reduced with Mos still performing at least as well as Rot overall (Figure 9). This result is intuitive, since the Rot strategy requires periodically reintroducing the already ineffective insecticide $\textrm{I}_{1}$ at full (100%) coverage during certain update periods. This structural feature explains why Rot consistently underperforms compared to Mos in our framework.

This configuration stand in contrast to those of Levick et al. (2017), who found that sequential deployment (Rot) outperformed mixtures (Mos) in some configurations. The apparent discrepancy arises from at least two main differences in initial assumptions. The first key distinction from Levick et al. (2017) lies in the initial efficacy assumptions regarding insecticides at the time of deployment. While authors in Levick et al. (2017) considered scenarios in which both insecticides retained effectiveness against mosquito populations, our framework assumes that insecticide $\textrm{I}_{1}$ has already lost efficacy due to widespread resistance, leaving $\textrm{I}_{2}$ as the sole insecticide with residual activity at deployment. The second difference pertains to the representation of the resistance trait: here, it is modeled as a scalar continuous variable $x \in \mathbb {R}$, rather than as a two-dimensional vector $x = (x_1, x_2) \in \mathbb {R}^2$, where $x_1$ and $x_2$ correspond to resistance levels against $\textrm{I}_{1}$ and $\textrm{I}_{2}$, respectively. This simplification reflects the notion that multi-insecticide resistance phenotypes often collapse onto low-dimensional trade-off axes or manifolds, a perspective that is plausible under at least two scenarios: when $\textrm{I}_{1}$ and $\textrm{I}_{2}$ represent different formulations of the same chemical compound, or when the resistance trait encapsulates a generalized mechanism effective against both insecticides.

Although evidence supporting such dimensionality reduction exists in the context of multidrug resistance (e.g., Barbosa et al. (2019); Imamovic and Sommer (2013); Lázár et al. (2014); Podnecky et al. (2018); Sakenova et al. (2024)), this may not universally apply to multi-insecticide resistance. Importantly, extending the trait space to $x \in \mathbb {R}^n$ does not fundamentally alter the main theoretical results established here, as demonstrated in Djidjou-Demasse et al. (2017). However, a significant challenge arises in specifying the age- and trait-dependent eggs-laying rate function $r(a,x)$. For instance, when considering two insecticides, this function generalizes to $r(a, x_1, x_2)$, and formulating an explicit expression without imposing a collateral insecticide resistance score $s$, which maps the multidimensional trait $(x_1, x_2) \in \mathbb {R}^2$ onto a unidimensional axis $s(x_1, x_2) \in \mathbb {R}$, remains a critical obstacle when modeling resistance as a multidimensional trait.

More than two insecticides may be available at the time of deployment. The model developed here naturally accommodates the introduction of additional insecticides into the landscape. For example, in Appendix A, we consider the case where two insecticides, $\textrm{I}_{2}$ and $\textrm{I}_{3}$, are introduced after a prolonged period of exclusive use of $\textrm{I}_{1}$. Assuming insecticide $\textrm{I}_{3}$ is more effective than $\textrm{I}_{2}$ at the time of deployment, the optimal mosaic strategy (Mos*) consists primarily of the exclusive use of $\textrm{I}_{3}$, followed by its gradual incorporation into the insecticide landscape (Figure S1). This finding suggests that the simultaneous deployment of two insecticides– differing only in their relative efficacy against the target vector population– may not represent the most effective strategy. Instead, a sequential approach, in which a first insecticide is used optimally until resistance reaches a critical threshold, followed by the introduction of the second insecticide, appears to yield greater benefits.

We also compared the simple mosaic (Mos) and simple rotation (Rot) strategies using insecticides $\textrm{I}_{2}$ and $\textrm{I}_{3}$. When the Rot strategy begins with $\textrm{I}_{2}$, and for high mutational variance ($\textrm{var}_0$), Mos consistently outperforms Rot regardless of the mutational variance ratio ($\textrm{var}$), with the performance gap widening as the insecticide update interval ($\mathrm{D{ur}}$) increases (Figure S2A, D). For $\textrm{var}_0 = 0.002$, Mos performs worse than Rot when $\mathrm{D{ur}} = 2$ or 3 years, but better when $\mathrm{D_{ur}} = 1$ year (Figure S2B, E). A similar qualitative pattern is observed for $\textrm{var}_0 = 0.001$, though with larger performance differences (Figure S2C, F). Notably, increasing $\mathrm{D_{ur}}$ slightly amplifies the performance gap ($\Delta (\text {Mos},\text {Rot})$) when $\textrm{var}_0 = 0.01$, but reduces it at lower mutational variances. When the Rot strategy begins with $\textrm{I}_{3}$ (Figure S3), Mos and Rot achieve nearly identical relative gains at the highest mutational variance ($\textrm{var}_0 = 0.01$), irrespective of $\mathrm{D_{ur}}$ or $\textrm{var}$. For lower $\textrm{var}_0$ values, however, Mos consistently outperforms Rot, and increasing $\mathrm{D_{ur}}$ further amplifies the performance gap ($\Delta (\text {Mos},\text {Rot})$) across all values of $\textrm{var}$.

Our results also reinforce a well-established principle in the epi-evolutionary management of insecticide resistance: the optimal dose for slowing resistance evolution often corresponds to the lowest dose that remains epidemiological effective. Similar patterns have been documented in antimicrobial stewardship, pesticide use, and pathogen evolution, where overly aggressive interventions intensify selection and accelerate the spread of resistance (e.g., Blanquart et al. (2017); Day and Read (2016); South and Hastings (2018)). In practice, however, deliberately “holding back” from maximal pressure is difficult to implement. Such strategies may conflict with short-term control objectives, require coordination across programs, and rely on robust surveillance and compliance. Our findings therefore highlight a persistent tension: although evolutionary theory shows that moderated intervention can outperform maximal pressure in the long run, translating this insight into operational policy remains challenging. This underscores the need for decision-support tools and governance frameworks that jointly optimize immediate disease-control goals and long-term resistance management.

Although the model developed here could be extended to explicitly incorporate additional mosquito life stages (e.g., larval and pupal stages) and thereby accommodate interventions such as larviciding or pupaciding, our analysis focuses on control measures targeting adult mosquitoes, notably insecticide-treated nets (ITNs) and indoor residual spraying (IRS). According to WHO guidelines, ITNs and IRS are classified as core malaria vector-control tools because they provide strong individual protection and substantially reduce adult mosquito survival, generating major community-level impact (Guidelines for Malaria Vector Control 2019). By contrast, larval source management (including larvicides and pupacides) is considered only a supplementary intervention, recommended solely in settings where larval habitats are few, fixed, and easily identifiable, and not as a replacement for ITNs or IRS. This reflects WHO’s conclusion that larviciding produces weaker and less consistent effects on malaria transmission (Guidelines for Malaria Vector Control 2019).

Another limitation of the model proposed here is the absence of climatic drivers–such as temperature, humidity, rainfall, and wind–which are known to strongly influence mosquito life-cycle traits and, consequently, population abundance, e.g., Abdelrazec and Gumel (2017); Djidjou-Demasse et al. (2020). Incorporating such environmental forcing would require coupling non-local interactions with a non-autonomous dynamical system, which raises substantial analytical challenges. Addressing this limitation therefore constitutes an important but technically demanding perspective for future work.

## Supplementary Information

Below is the link to the electronic supplementary material.

Supplementary file 1 (pdf 1225 KB)

## Data Availability

All the data used are included in this paper.

## Model with 3 insecticides

Here we assume that after the failure of $\textrm{I}_{1}$, we introduced two insecticides, denoted $\textrm{I}_{2}$ and $\textrm{I}_{3}$. Insecticide $\textrm{I}_{2}$ is characterized by a relative effectiveness compared to $\textrm{I}_{1}$ with values ranging in $\{0.5, 0.6, 0.7, 0.8\}$, while insecticide $\textrm{I}_{3}$ has a relative effectiveness compared to insecticide $\textrm{I}_{1}$ fixed to 0.9. During the $\overline{T}$ years period of insecticide deployment, the insecticide landscape composed of $\textrm{I}_{1}$, $\textrm{I}_{2}$, and $\textrm{I}_{3}$, is revised *N* times at times $T_0$, $T_1$, $\cdots$, $T_{N-1}$, at regular intervals of length $\mathrm{D_{ur}}$. Moreover, during each $n^{th}$ period, the insecticide landscape composition is defined by the proportions $p^n_1$, $p^n_2$ and $p^n_3$, representing the proportion of insecticides $\textrm{I}_{1}$, $\textrm{I}_{2}$ and $\textrm{I}_{3}$, respectively, with $p^n_1+p^n_2+p^n_3 \le 1$. The model with three insecticides is given for $n\in \{1,\cdots , N\}$ and $t\in [T_{n-1},T_n)$, by:

$$\begin{aligned} {\left\{ \begin{array}{ll} \displaystyle A_{0}^{n}(t,a=0,x)= \left( 1 - p_{1}^{n} - p_{2}^{n} - p_{3}^{n}\right) \tau (x) \sum _{j=0}^{3}\gamma _{j}(x)E_{j}^{n}(t,x) , \\ \displaystyle A_{1}^{n}(t,a=0,x)= p_{1}^{n} \tau (x) \sum _{j=0}^{3}\gamma _{j}(x)E_{j}^{n}(t,x), \\ \displaystyle A_{2}^{n}(t,a=0,x)= p_{2}^{n} \tau (x) \sum _{j=0}^{3}\gamma _{j}(x)E_{j}^{n}(t,x), \\ \displaystyle A_{3}^{n}(t,a=0,x)= p_{3}^{n} \tau (x)\sum _{j=0}^{3}\gamma _{j}(x)E_{j}^{n}(t,x), \end{array}\right. } \end{aligned}$$

and for $$j\in \{0,1,2,3\}$$:

$$\begin{aligned} {\left\{ \begin{array}{ll} \partial _t E_{j}^{n}(t,x)= H(E^{n}(t)) \int _{\Omega } \int _0^\infty m_{j}(x,y)r(a,y)A_{j}^{n}(t,a,y)\textrm{d}a\textrm{d}y - (\mu _{j}(x) + \gamma _{j}(x)) E_{j}^{n}(t,x), \\ \left( \partial _t + \partial _a \right) A_{j}^{n}(t,a,x)= - d_{j}(a,x) A_{j}^{n}(t,a,x), \end{array}\right. } \end{aligned}$$

together with the initial conditions $E_{j}^1(0,x) = E_{j,0}(x),$ $A_{j}^1(0,a,x) = A_{j,0}(a,x)$ and $E^n_j(T_{n-1},x) = E^{n-1}_j(T_{n-1},x),$ $A^n_j(T_{n-1},a,x) = A^{n-1}_j(T_{n-1},a,x)$ when $n \in \{ 2, 3, \ldots , N\}$.

Here, $E_{j}^{n}$ represents the total number of egg laid by AFM in *j*-insecticide state and $A_{j}^{n}$ denotes the total number of adult mosquitoes in *j*-insecticide state, at time period *n* and for $j\in \{0,1,2,3\}$. The rest of parameters are unchanged.
