# Supplementary material for: Optimizing insecticide deployment strategies to delay quantitative resistance in mosquito populations
Source: J Math Biol. 2026 Feb 23;92(3):37. doi: 10.1007/s00285-026-02343-z (PMC12926253; doi:10.1007/s00285-026-02343-z)
Supplement: Supplementary file 1 — (pdf 1225 KB) [file 285_2026_2343_MOESM1_ESM.pdf]

B Supplementary figure

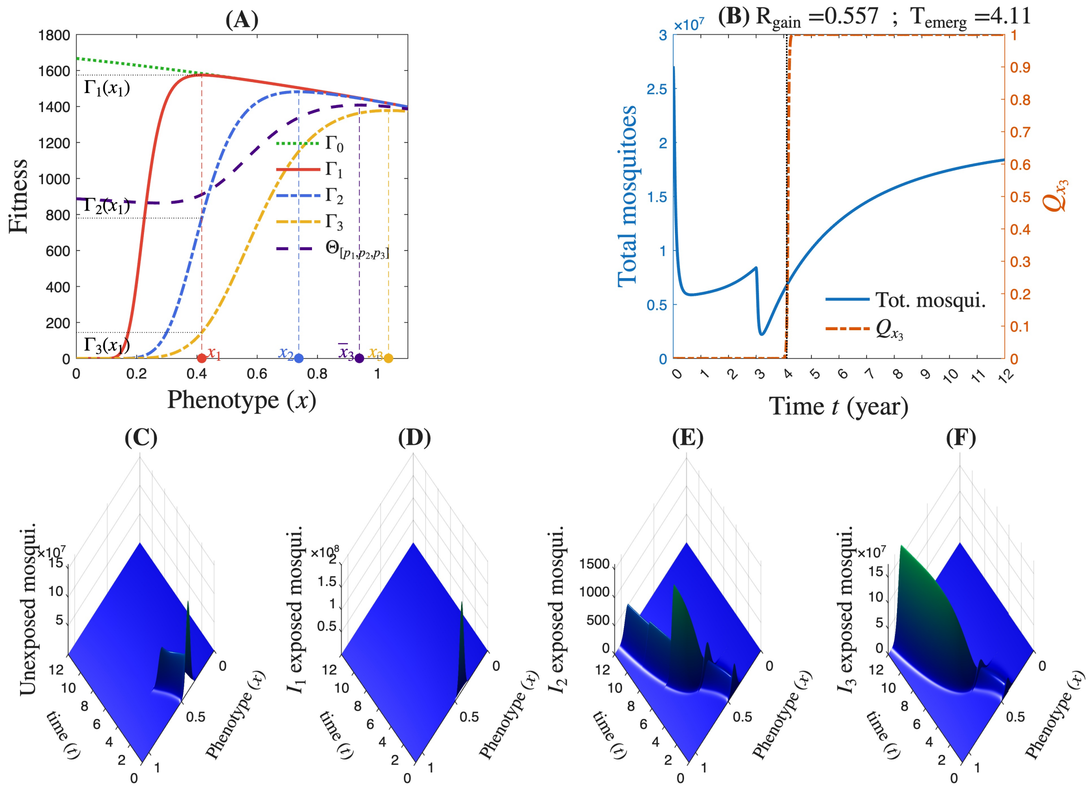

Figure S1: **Evolutionary dynamics with the optimal deployment strategy for a prior exposure rate of $p_0 = 0.6$, $\mathrm{var}_0 = 0.002$ and $\mathrm{r_{eff}} = 0.5$.** We established that the optimal mosaic strategy is obtained for $p_1 \approx (0, 0, 0, 0)$, $p_2 \approx (0, 0, 0, 0)$, $p_3 \approx (0.47, 0.99, 0.99, 0.99)$. **(A)** the fitness functions. **(B)** Dynamics of total population and population with phenotypic value x_3. **(C-F)** The evolutionary dynamics for unexposed and exposed adult mosquitoes.

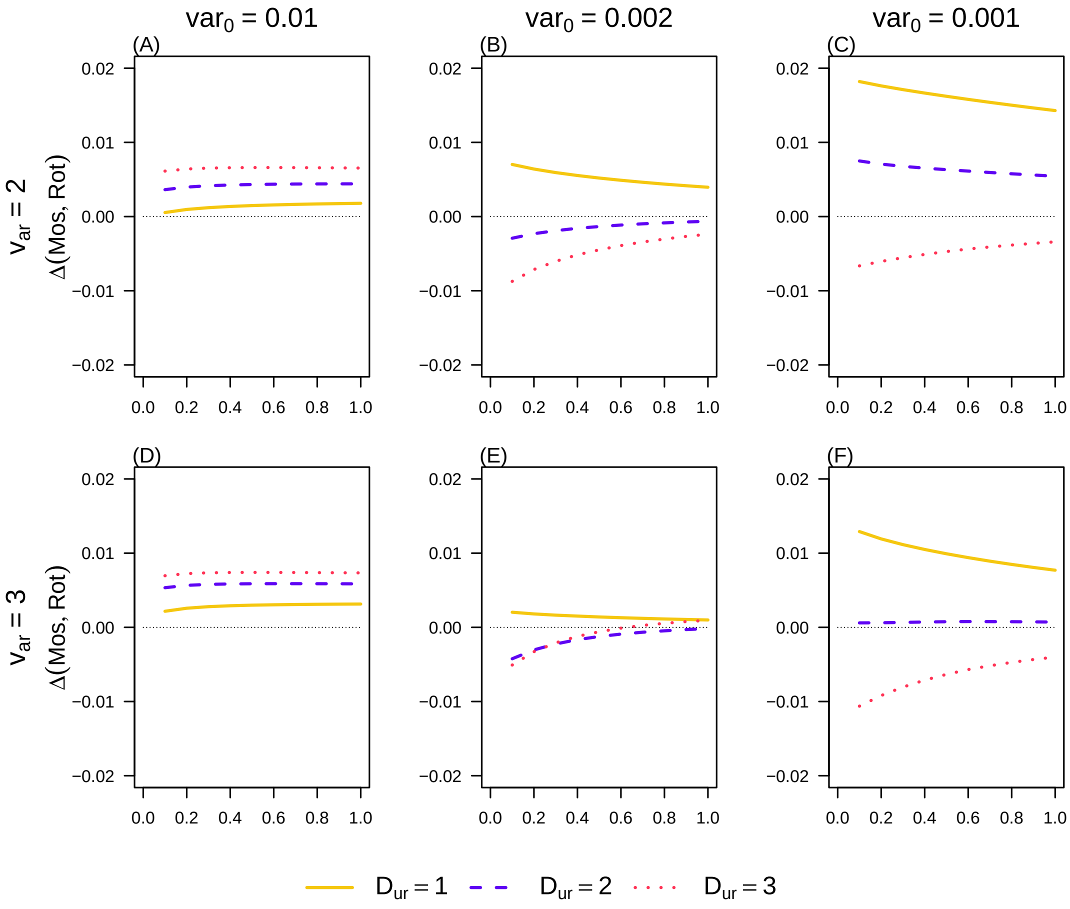

Figure S2: **The difference in performance between Mos and Rot strategies.** Here rotation starts with insecticide I_2 then I_3 and $\Delta(\text{Mos}, \text{Rot}) = R_{\text{gain}}(\text{Mos}) - R_{\text{gain}}(\text{Rot})$. The mutational variance (var_0) and the mutational variance ratio (var) are variables.

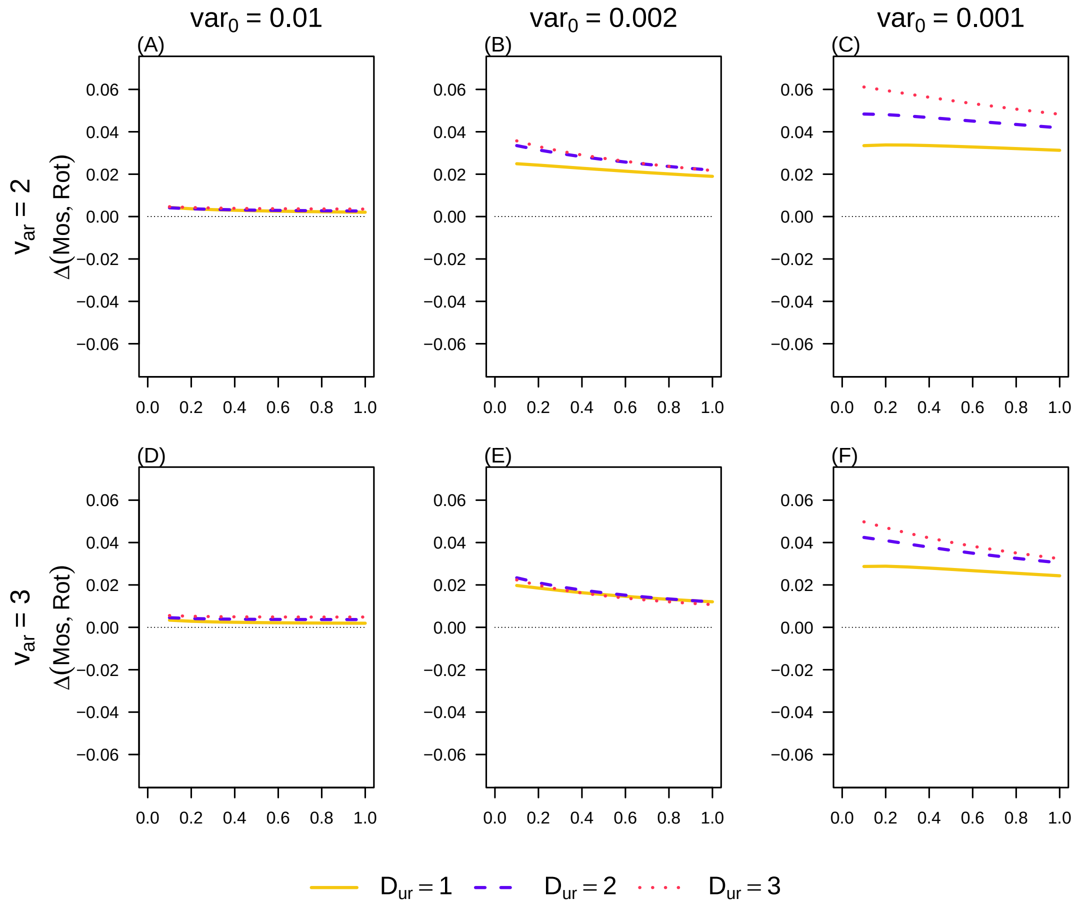

Figure S3: **The difference in performance between Mos and Rot strategies.** Here rotation starts with insecticide I_3 then I_2 and $\Delta(\mathrm{Mos}, \mathrm{Rot}) = R_{\mathrm{gain}}(\mathrm{Mos}) - R_{\mathrm{gain}}(\mathrm{Rot})$. The mutational variance (var_0) and the mutational variance ratio (var) are variables.
